# Long-Term Safety of Repeated Blood-Brain Barrier Opening via Focused Ultrasound with Microbubbles in Non-Human Primates Performing a Cognitive Task

**DOI:** 10.1371/journal.pone.0125911

**Published:** 2015-05-06

**Authors:** Matthew E. Downs, Amanda Buch, Carlos Sierra, Maria Eleni Karakatsani, Shangshang Chen, Elisa E. Konofagou, Vincent P. Ferrera

**Affiliations:** 1 Department of Biomedical Engineering, Columbia University, New York, New York, United States of America; 2 Department of Radiology, Columbia University, New York, New York, United States of America; 3 Department of Neuroscience, Columbia University, New York, New York, United States of America; Institute of Neurology (Edinger-Institute), GERMANY

## Abstract

Focused Ultrasound (FUS) coupled with intravenous administration of microbubbles (MB) is a non-invasive technique that has been shown to reliably open (increase the permeability of) the blood-brain barrier (BBB) in multiple *in vivo* models including non-human primates (NHP). This procedure has shown promise for clinical and basic science applications, yet the safety and potential neurological effects of long term application in NHP requires further investigation under parameters shown to be efficacious in that species (500kHz, 200–400 kPa, 4–5μm MB, 2 minute sonication). In this study, we repeatedly opened the BBB in the caudate and putamen regions of the basal ganglia of 4 NHP using FUS with systemically-administered MB over 4–20 months. We assessed the safety of the FUS with MB procedure using MRI to detect edema or hemorrhaging in the brain. Contrast enhanced T1-weighted MRI sequences showed a 98% success rate for openings in the targeted regions. T2-weighted and SWI sequences indicated a lack edema in the majority of the cases. We investigated potential neurological effects of the FUS with MB procedure through quantitative cognitive testing of’ visual, cognitive, motivational, and motor function using a random dot motion task with reward magnitude bias presented on a touchpanel display. Reaction times during the task significantly increased on the day of the FUS with MB procedure. This increase returned to baseline within 4–5 days after the procedure. Visual motion discrimination thresholds were unaffected. Our results indicate FUS with MB can be a safe method for repeated opening of the BBB at the basal ganglia in NHP for up to 20 months without any long-term negative physiological or neurological effects with the parameters used.

## Introduction

The blood brain barrier (BBB) is a highly selective biological system that maintains brain homeostasis [1]. Due to its high efficiency, the BBB prevents 99% of currently available small molecules (> 400 Da) and all large molecule drugs from crossing the BBB [2]. This hinders clinical treatment of neurological diseases and disorders, as well as development of novel drugs to treat the aforementioned diseases [3],[4]. Current techniques for drug delivery through the BBB are either invasive and localized or non-invasive with regionally nonspecific delivery [5],[6],[7]. An optimal system for trans-BBB drug delivery would be non-invasive, have high regional specificity and be reproducible without permanent long-term neurological effects.

Our group and others have shown over the past decade that focused ultrasound (FUS) with microbubbles (MB) is an effective technique to open the BBB for multiple *in vivo* animal models [8],[9],[10],[11]. This technique is performed noninvasively and the opening closes within hours to days depending on the acoustic pressure used [12],[13]. FUS mediated BBB opening has been shown to be effective for facilitating drugs such as doxorubicin to treat tumors and assist delivery of other small molecules (gold nanoparticles, brain-derived neurotrophic factor, adeno-associated virus) across the BBB [14],[15],[16],[17]. FUS with MB is a promising technique for targeted drug delivery in the central nervous system, but before clinical application of this technique in humans can occur, the long term effects of the technique on behavioral and cognitive function must be further investigated.

The introduction of MB coupled with lower-pressure FUS has been shown not to damage tissue or cause neurological deficits in mice [18],[19]. Our group and others have shown that for specific parameters the FUS with MB procedure can be safe for non-human primates (NHP) [20],[21]. The primary method of evaluating potential damage from FUS BBB opening in NHP is MRI, specifically T2-weighted and Susceptibility-Weighted Imaging (SWI) scans [20]. There has been one study utilizing histological evaluation of the FUS BBB opening procedure in NHP over a time frame of 2–26 weeks [21]. While MRI and histology are useful for detecting cellular damage from the procedure, neither method can detect if the FUS with MB procedure has an effect on neurological function. A previous study reported the effects of several weeks of FUS with MB application on the thalamus (lateral geniculate nucleus) in the NHP model using a visual acuity task [21]. To date there has not been any study conducted on the neurological effects on motor and cognitive processing of repeated (> 13 months) FUS with MB procedures with BBB opening in NHP.

In the current study, we investigated the effects of repeatedly applying FUS to the basal ganglia of four NHP over time frames ranging from 4 to 20 months. Acoustic pressures for the FUS with MB procedure were varied through the initial part of the experiment to determine safe ranges of these parameters in the basal ganglia. Within the basal ganglia, the caudate and putamen regions were selected as targets for FUS BBB opening as they are both implicated in voluntary motor control, goal-directed action, memory, learning, and decision-making, and are affected in Parkinson’s Disease [22],[23],[24],[25]. The FUS with MB procedure was applied to NHP under general anesthesia using a stereotaxic targeting system. Constant monitoring of vital signs (respiration, blood pressure, heart rate, blood oxygenation) before, during, and after the FUS with MB procedure was used to evaluate any potential physiological changes from repeated procedures. Following each FUS with MB procedure, the safety of the procedure (lack of edema or hemorrhage) was evaluated with T2-weighted MRI and SWI sequences. The BBB opening was verified with contrast enhanced 3D T1-weighted MRI sequences. The safety of the FUS with MB procedure was also investigated with behavioral assessment using a reaching task based on a combination of a reward magnitude bias (RMB) paradigm and a random dot motion (RDM) task [26],[27],[28]. This combined task tested visual perception, decision-making, motivation and motor function to determine if the BBB opening affected known neurological pathways of the putamen [22],[29]. The behavioral evaluation coupled with the MRI results establish a multi-faceted approach for verifying safety of long term FUS BBB opening in NHP.

## Methods

### Subjects and Ethics Statement

All NHP procedures described herein were approved by the Institutional Animal Care and Use Committees of Columbia University and the New York State Psychiatric Institute. Adult male macaques (n = 4) were used in all experiments (ages 8–20 years, weights 5–9 kg); one Macaca fascicularis (NHP A) and three Macaca mulatta (NHP O, Ob, N). The NHP were housed (N & O were paired with each other) in a room with a 12 hour light dark cycle. NHP were housed in a 3 ft^3^ Erwin-Steffes Enhanced Environmental Housing System (Primate Products Inc., Immokalee, FL, USA) and given access to play cages (total area 3 ft^2^ x 7 ft) with various enrichment toys (wooden food logs, plastic chew stars, mirror balls). They were fed constant rations of vitamin enriched dry primate biscuits and given 1L of water on days when they were not tested behaviorally. On testing days, the NHP performed the behavioral task for water reward until they were satiated. NHP were not given additional fluids even if they did not work for a full liter as supplementing the water they received from working with ‘free’ water would reduce their motivation to perform the behavioral task. Each day, after the behavioral session was completed, they were given a fruit treat.

For each FUS with MB procedure, NHP were initially sedated with ketamine (10–12 mg/kg) and given a dose of atropine (0.04mg/kg). An endotracheal catheter was inserted, after which the NHP were anesthetized with isoflurane (1–2%) mixed with O_2_ (2 L/min) for the duration of the procedure. A stereotactic frame (David Kopf Instruments, CA, USA) was used for head fixation to ensure accuracy of the FUS targeting. The scalp was shaved and depilatory cream removed remaining hair to reduce interference with the acoustic transmission. A catheter was placed in the saphenous vein for IV delivery of 0.9% saline, MB and the MRI contrast agent gadodiamide (Omniscan, 573.66 DA, GE, Healthcare, Princeton, NY, USA). A heated water blanket was used to maintain body temperature during the FUS with MB procedure. Heart rate (EKG), blood oxygenation (SpO_2_), end-tidal CO_2_ expiration, respiratory rate, and non-invasive blood pressure were recorded during the procedure. Five time-points were used for analysis: immediately after the NHP was placed in the stereotax, 30 seconds prior to MB injection, 60 seconds into the sonication, 30s after sonication, and the last point at the end of the procedure when the NHP was taken out of the stereotax.

### FUS with MB procedure

All MB used in the procedure were made in-house and centrifuged for size isolation with a mean MB diameter of 4–5μm [30]. A 500-kHz center frequency focused ultrasound transducer was used for all experiments (H-107, Sonic Concepts, WA, USA). The built-in water bladder system on the transducer was filled with de-ionized water and circulated through a degasser (Sonic Concepts, WA, USA) for at minimum of 30 minutes before the FUS with MB procedure. Acoustic pressures ranging from 200–400 kPa were applied with a pulse length of 10ms, pulse repetition frequency of 2 Hz with a total sonication duration of 2 minutes per target location. The caudate and putamen regions of the basal ganglia were selected as the two main targeting regions. A detailed list of acoustic pressures and targeted locations for each NHP are located in Table 1. The transducer was mounted on a 9-degrees-of-freedom stereotactic arm (David Kopf Instruments, CA, USA) that was attached to the stereotactic frame securing the head of the NHP. Stereotactic coordinates were found with an in-house targeting algorithm calibrated for the focal distance of our transducer [20]. An initial six-second sonication without MB was used as a control for real time cavitation monitoring [31]. A bolus injection of MB with a concentration of 2.5^8^ MB/kg was used for each initial sonication. The FUS with MB procedure was initiated at the onset of IV MB injection with an average circulation time of 10s before MB reached the focal area. Real-time monitoring via a hydrophone (Y-107, Sonic Concepts, WA, USA) was used to verify that MB had reached the focal zone and to monitor harmonic, ultraharmonic and inertial cavitation levels [31]. The hydrophone was placed through a center hole in the FUS transducer allowing overlap of their focal regions. A pulse generator (Olympus, PA, USA) drove the initial signal which was passed through a 20-dB amplifier (5800, Olympus NDT, Waltham, MA, USA) providing the signal to the transducer. Output from the hydrophone was filtered through a pulse-receiver (5072PR, Olympus, PA, USA) before being digitized (Gage Applied Technologies, Inc., Lachine, QC, Canada) and recorded. For some experiments (n = 31), a second sonication was conducted < 1 min after first sonication at an area adjacent (average 1.5 cm shift on anterior-posterior axis) from the initial sonication location, but still within the same targeted subcortical nuclei. For those sonications, real-time monitoring verified that MB remained in circulation and had not yet been filtered out, thus a second injection of MB was not necessary. If the caudate and the putamen were both sonicated on the same day, a 20-min waiting period occurred between sonications allowing the MB to be filtered from the bloodstream. Once the 20 minutes passed, another negative control was acquired to verify the MB had been filtered before the second bolus injection and sonication occurred.

**Table 1:** Targets and acoustic pressures of FUS with MB procedures for each NHP.

NHP	Brain Target	Acoustic Pressures (kPa)	Duration
A	L. Putamen	300 (n = 12)	10 mo*
N	L. Putamen	200 (n = 1), 275 (n = 8)	11 mo
N	R. Putamen	400 (n = 9)	10 mo*
N	L. Caudate	250 (n = 6), 300 (n = 3)	12mo
O	L. Putamen	250 (n = 4), 275 (n = 6)	12 mo
O	L. Caudate	200 (n = 4), 250 (n = 3), 275 (n = 1)	20 mo
Ob	L. Putamen	400 (n = 4)	4 mo*

The n indicates the number of time FUS was applied to that region at that pressure. The duration is the amount of time over when the FUS with MB procedures occurred. Asterisk durations indicate time while completing RDM + RMB task.

### MRI Analysis

MRI scans (3T, Philips Medical Systems, MA, USA) for each NHP were acquired either 30 minutes (n = 36) or 30 hours (n = 25) after the FUS with MB procedure. T2-weighted sequences (TR = 10ms, TE = 27ms, flip angle = 90°, spatial resolution = 400 x 400 μm^2^, slice thickness = 2mm with no interslice gap) and 3D Susceptibility-Weighted Image (SWI) sequences (TR = 19ms, TE = 27ms, flip angle = 15°, spatial resolution = 400 x 400 μm^2^, slice thickness = 1 mm with no interslice gap) were used to verify safety of the procedure. 3D Spoiled Gradient-Echo (SPGR) T1-weighted sequences (TR = 20ms, TE = 1.4ms, flip angle = 30°, spatial resolution = 500x 500 μm^2^, slice thickness = 1mm with no interslice gap) were acquired after IV administration of gadodiamide at a dose of 0.2 ml/kg to confirm BBB opening. Gadodiamide was selected as the contrast agent as it does not cross the intact BBB. There was a 30-min diffusion period after IV administration of gadodiamide before the post T1-weighted (T1-Post) scan was acquired. On a separate day when the BBB was not open (no FUS with MB procedure for > 1 week), another contrast enhanced T1-weighted sequence was acquired for post-processing purposes (T1-Gado). Each T1-Post sequence was post-processed to find the opening location and the volume of the induced BBB opening. The T1-Post and T1-Gado sequences were aligned to a high resolution stereotaxically oriented T1-weighted sequence for each NHP with the FSL toolbox [32]. A grey and white matter segmentation map of each brain was generated and used to find the average voxel intensity of the grey and white matter for image normalization of the T1-Post and T1-Gado sequences. Subsequently, the normalized T1-Post was divided by the normalized T1-Gado to locate of voxels where the contrast was increased over baseline. Voxels that had a contrast increase of 10% within the focal region were counted to determine the volume of the BBB opening. The center of the opening was found by weighing each voxel above the threshold with its intensity and then averaging all weighted voxel locations. Contrast enhanced T1-weighted MRI scans were also acquired at least one week after any FUS with MB procedures at the midpoint and after the final FUS with MB procedure for each animal. These scans were processed using the aforementioned pipeline to verify closure of the BBB in the targeted regions.

### Behavioral Testing

**Fig 1:**
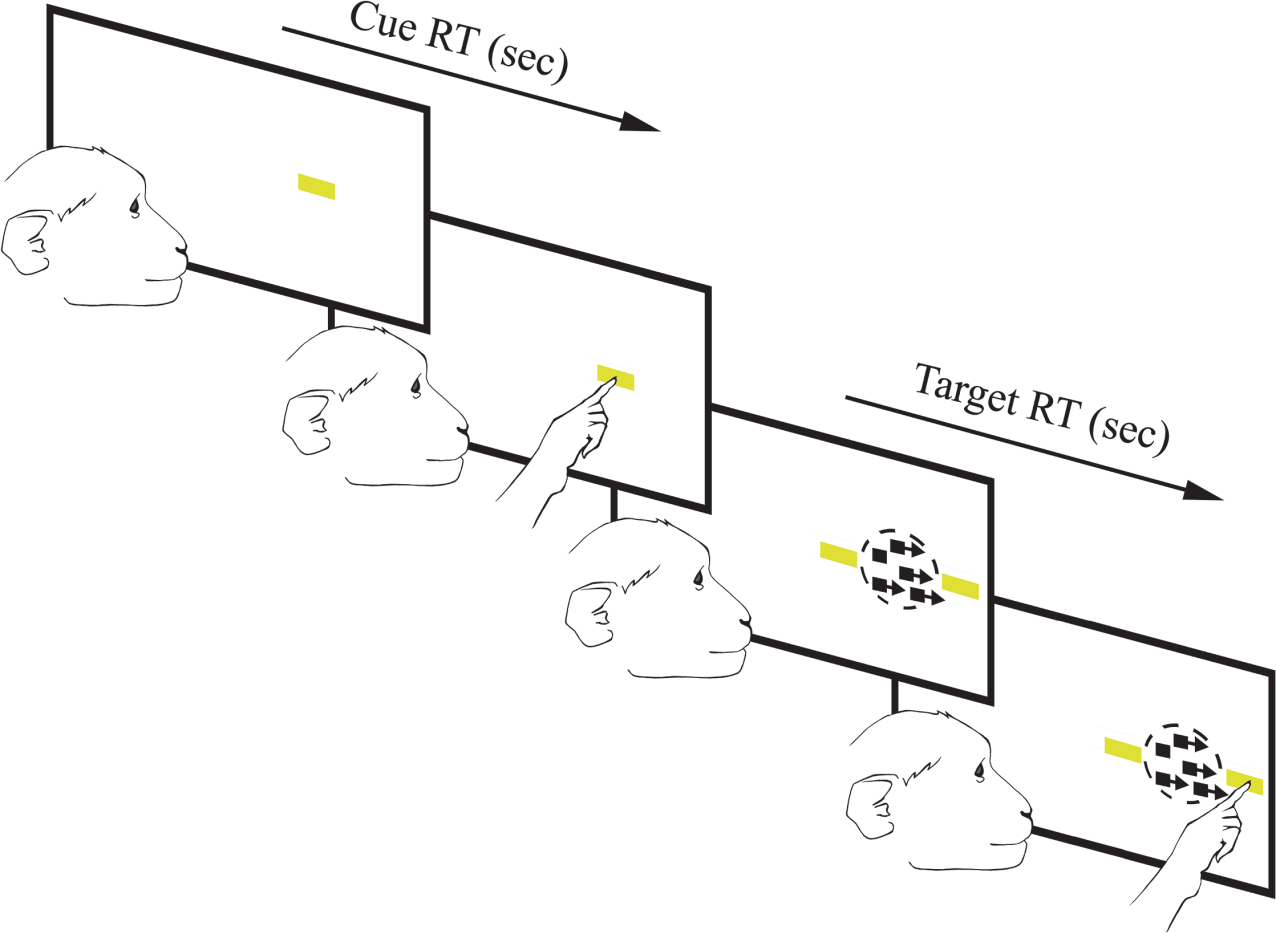
Random dot motion and reward magnitude bias task. Cues appear in the center of the left or right side of the screen randomly. Once the initial cue is touched three new stimuli appear: A correct target appears on either the inside (towards the middle of the screen) or the outside (towards the edge of the screen) on the same side the intial cue, and a distractor target will appear opposing the correct target as well as moving dots will appear where the initial cue was. Horizontal and vertical bars indicate high and low reward respectively. Reward magnitude is random so subjects cannot predict reward order.

Three of the NHP (A, N, Ob) were trained to touch visual stimuli presented on a 20-inch color LCD touchpanel monitor (NEC 2010X with 3M SC4 touch controller, Fig 1). Each successful trial was rewarded with 1 or 5 drops of water delivered through a spout positioned in front of the mouth. Daily sessions ranged between 1–3 hours during which the NHP would typically perform 700–2000 trials of the task and receive 100–400 ml of fluids. At the start of each session, the NHP was placed in an IACUC-approved chair in which their head and both hands were free to move. A vertical divider was placed between the chair and the monitor to prevent the NHP from reaching across the display. Thus, stimuli presented on the right side of the monitor were only accessible to the right hand, and likewise for the left side.

The reaching task combined the well-established Random Dot Motion detection paradigm (RDM) with a Reward Magnitude Bias (RMB) [28],[29],[30]. The combined task tested reaction time (RT), touch error (TE), motivation and decision making. For each trial an initial cue, a horizontal or vertical yellow bar was presented randomly on either the left or right side of the screen (the pixel area and intensity was the same for both orientations). A horizontal bar signified a large reward of five drops of water while a vertical bar signified a small reward of one drop of water (5:1 reward bias). Previous studies implementing a RMB paradigm have used smaller reward differentials, but we selected a larger bias to make the difference more salient to the animals and thereby increase the likelihood or magnitude of any effect of the FUS with MB procedure on motivation.

Once the NHP touched the initial cue, the display was updated to show a random dot motion stimulus and two secondary targets of the same shape, color and intensity as the cue. The dots appeared in a circular aperture. A percentage (0–70%, step size of 10%) of the dots moved coherently toward one target while the remaining dots moved randomly [30]. If the NHP touched the target towards which the dots were moving, the response was scored as correct and the NHPs. received the amount of water reward corresponding to the orientation of the targets. If the NHP touched the other target (the “distracter”), the response was scored as incorrect and no reward was given. If the NHP touched any other part of the display or failed to touch the display altogether, the trial was scored as a failure and was not rewarded.

Each trial took a maximum of 4 seconds, and each stage of the trial had a time limit for the NHP to respond or the trial would reset. The initial cue was present on the screen for a maximum of 1.5 seconds, and the dots with the targets were on the screen for a maximum of 2.5 seconds. Trials that were ignored or aborted would be recycled and presented again. Reaction time (RT) to the initial cue was defined as the time from the visual onset of the cue until the first touch registered by the touchpanel screen. RT to the correct target or distracter was defined as the time from the onset of the moving dots until the subsequent touch registered by the touchpanel.

All NHP were full trained on the task (accuracy at discriminating dot direction > 75%) prior to recording. NHP O was excluded from this portion of the study as it did not achieve criterion performance on the RDM task.

### Data Analysis

The data were examined with two separate pipelines: The first examined the data sequentially over the duration of the experiment. The second divided the data into groups depending on the day of acquisition relative to the day of the FUS with MB procedure (-1, 0, 2, 3, 4 and 5+ days).

For the first pipeline the mean raw reaction time (RT) were sorted by day, and response (to either the initial cue or correct target). A one-way ANOVA was used to detect significant variance within each NHP across the duration of the experiment. Touch error (TE) to the cue and target were also analyzed using this pipeline. TE was defined as the distance between where the NHP touches the screen and the center of the cue or target stimulus

For the second method, days -1 and 5+ were considered to be a baseline since previous work has shown that the BBB openings created with the pressures being applied with this study close within three days [12]. This gives an additional two-day buffer to ensure the BBB has completely closed. A one-way ANOVA with Tukey’s HSD criterion (p < 0.05) was used for analysis of the RT and TE between groups to the initial cue and correct target. These groups were further divided according to which hand was used to respond and reward level. The average difference and confidence interval between low and high reward as well as ipsilateral and contralateral hand for RT and TE was found for each day. Days were determined to have significantly different means to day 0 if the 95% confidence interval of the mean did not overlap with the 95% confidence interval of day 0.

Random dot motion accuracy was divided into groups using the same conditions as in the second method described above. The performance accuracy (percentage of correct trials) from each group was sorted by coherence level and fit with a psychometric curve (Naka-Rushton). Psychophysical threshold for detecting direction of motion was determined as the coherence level corresponding to 80% correct responses.

## Results

### Vital Signs

**Fig 2:**
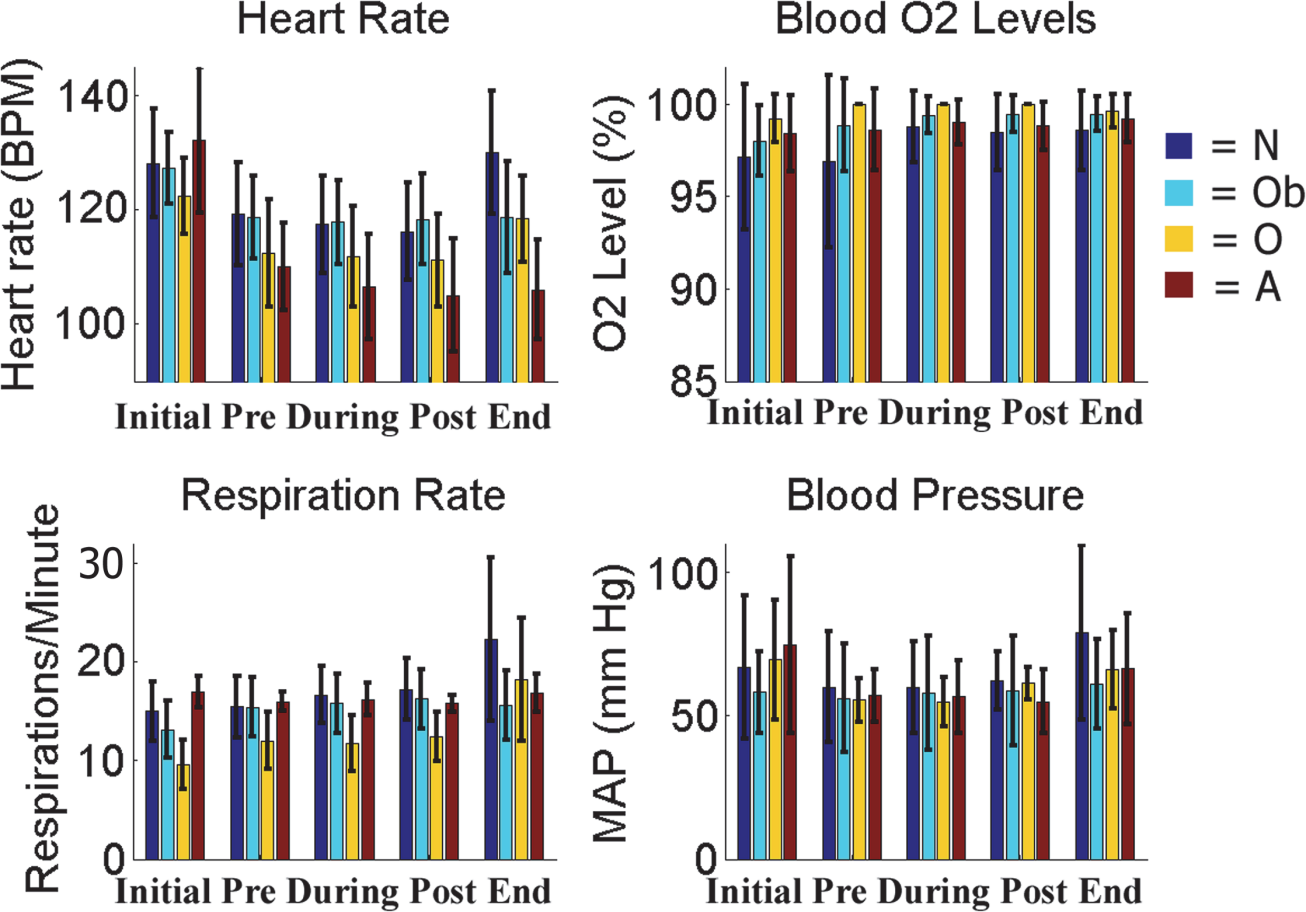
Vital Monitoring. Vitals collected at five points throughout the duration of the FUS with MB procedure. Results in the grey shaded region indicate time-points acquired during the FUS proceure.

Throughout the duration of the FUS with MB procedure the NHP vital signs were monitored and recorded. There were no significant differences between the recorded values for all the NHP in this study and previously published data for average vitals of macaques under isoflurane for heart rate and mean arterial pressure (student t-test, p < 0.05) [33]. Fig 2 shows the average heart rate, blood O^2^ levels, respiration rate and blood pressure throughout the FUS with MB procedure. There was no significant difference between vital recordings with the same NHP when either the targeting region or the acoustic pressure changed (one-way ANOVA, p < 0.05). Once the NHP regained full consciousness (3 hours after the FUS with MB procedure), no qualitative differences in their behavior within the husbandry room was observed. NHP would return to normal locomotion, eating, drinking and in the case of N and O, normal social behavior (grooming, playing). The weight for all NHP stayed consistent across the study. The only decrease occurred on days after there was no behavioral testing due to a restriction of fruit rewards since testing did not occur. Food and water intake stayed consistent and the fluctuations were not significant when compared to a control NHP not being used in the FUS with MB procedures housed in the same facility (one-way ANOVA, p < 0.05).

### MRI Analysis

**Fig 3:**
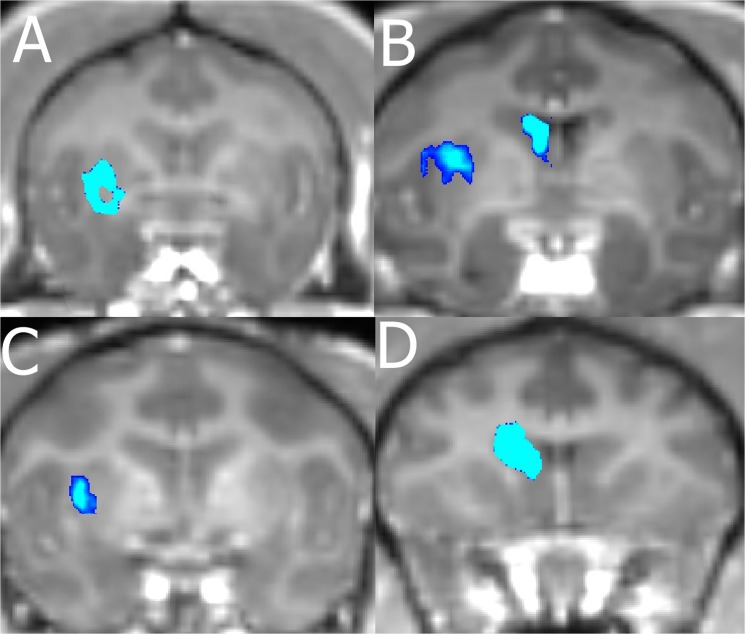
Contrast Enhanced BBB opening. The blue region shows a 10% increase in contrast over the background. A) Opening in the putamen of A. B) Opening in the caudate and putamen of N. C) Opening in the putamen of Ob. D) Opening in the caudate of O

**Fig 4:**
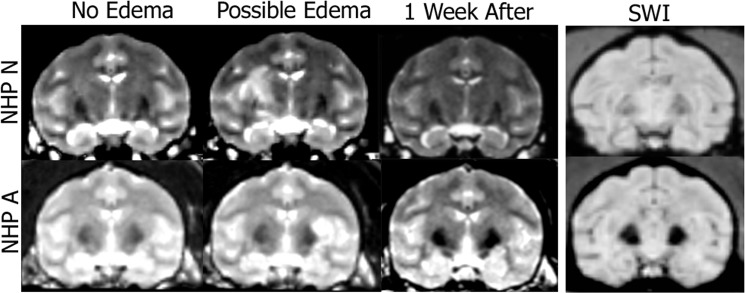
T2-weighted MRI and SWI scans of NHP N and A. T2-weighted sequences can be used to detect potential edema. The first column shows no atypical hyperintense voxels in the target region. The second column shows atypical hyperintense voxels in the target region. The third column verifies the atypical hyperintense voxels from the previous week were no longer present. The fourth column shows the SWI scans from the day when hyperintense voxels were detected on the T2 scan (acquired the same day as column 2).

We were able to verify BBB opening in 98% of our FUS with MB procedures by comparing each NHP T1-post with the respective T1-Gado. Table 2 shows the number of times opening was achieved per location in each NHP. A typical representation of size and location of openings for the NHP is presented in Fig 3. Average volume of opening was 203 mm^3^ with an average focal shift of 3 mm between the center of BBB opening and the planned target region. Table 3 lists the sizes of openings per NHP for each targeted area. No damage (hemorrhage or edema) was observed in the T2-weighted and SWI scans for the majority of the NHP using pressures in the range 200–400 kPa with 4–5μm MB (n = 57). There were four cases where the T2-weighted scans exhibited hyperintense voxels in the area of targeting indicating possible edema. These occurred with NHP N and A only. These cases appeared on the final FUS with MB procedure for N, and the final 3 procedures for A. Fig 4 shows T2-weighted scans from two of the four cases with hyperintense voxels in the target area as well as T2-weighted scans one week later showing no hyperintense voxels in the target area. No hyper- or hypointense voxels were detected in the target region on the SWI scans for all NHP.

**Table 2:** Targets and BBB openings of FUS with MB procedures for each NHP and duration of RDM + RMB task.

NHP	Target	# of FUS	BBB opening	Duration of Task
A	L. Putamen	12	12	13 mo
N	R. Putamen	9	8	15 mo
Ob	L. Putamen	4	4	4 mo

**Table 3:** Volume of BBB opening per NHP and location.

NHP	Brain Target	Acoustic Pressures (kPa) and Volume of Opening (mm^3^)
A	L. Putamen	300: 494 ±185
N	L. Putamen	200: 704, 275: 118 ±18
N	R. Putamen	400: 29 ±23
N	L. Caudate	250: 177 ± 182, 300: 326
O	L. Putamen	250: 1792± 1447, 275: 2480±1550
O	L. Caudate	200: 459 ± 61, 250:1438 ± 823, 275: 220
Ob	L. Putamen	400: 418 ± 347

Table shows the BBB opening volume per target location, animal and pressure.

### Behavioral Task

Three NHP (A, N, Ob) were tested on the RDM + RMB task outlined in the methods section which recorded RT, TE, motivation and decision making. Throughout the 4–15 month duration of the experiment, FUS with MB procedures were conducted targeting the putamen as shown in Table 2. RT for the initial reach to the cue is a simple reaction time. RT for the second reach to the target is a choice reaction time, as the NHP must choose between the correct and incorrect targets.

**Fig 5:**
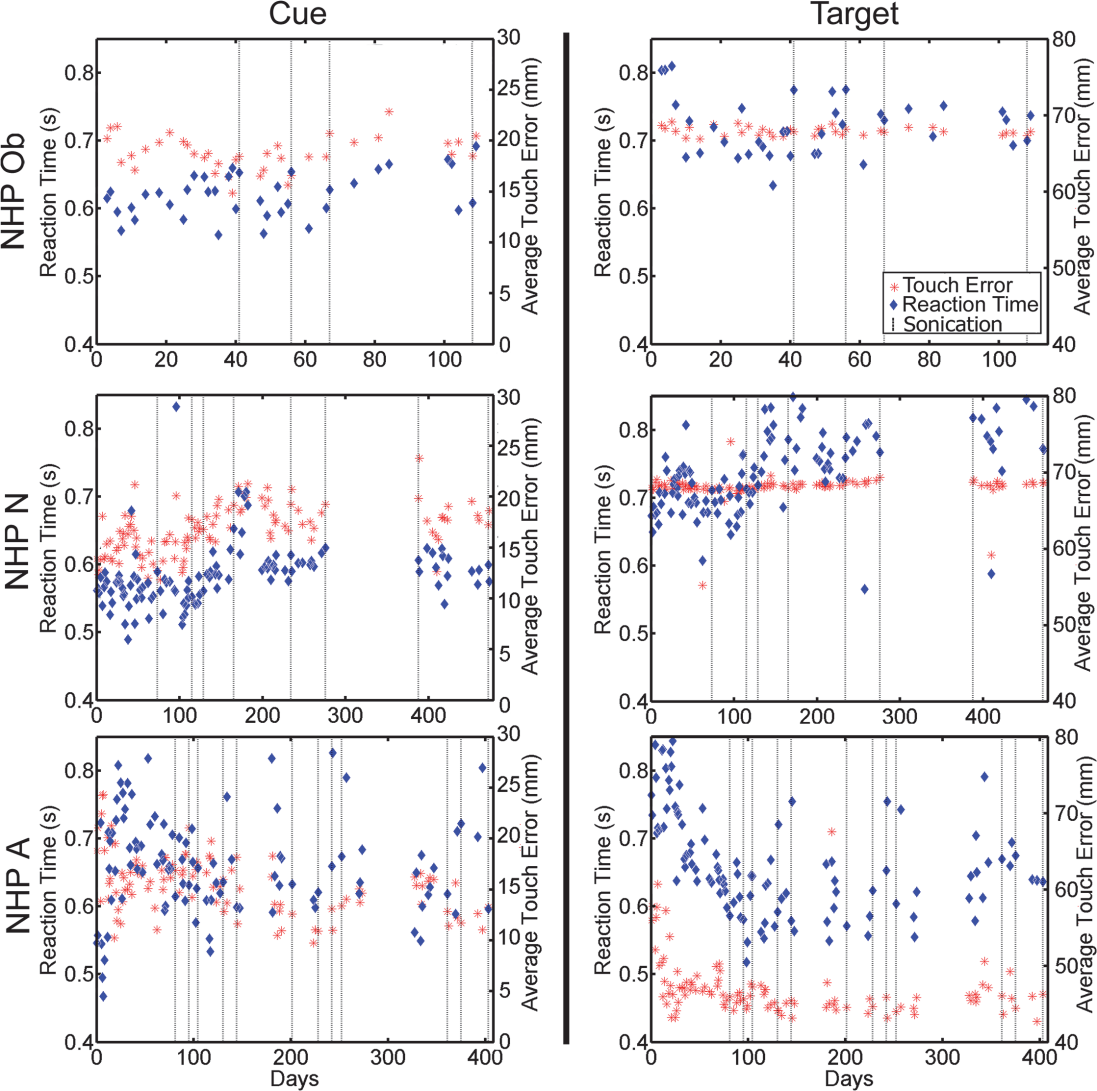
Raw reaction time and touch error to initial cue (left column), and to the correct target (right column). Blue diamonds indicate raw reaction time in seconds to either the initial cue or correct target. Red asterisks indicate raw touch error to either the initial cue or correct target. Reaction time is plotted on the left vertical axis while touch error is plotted on the right vertical axis. Dashed vertical lines indicate days when the FUS with MB procedure occurred.

The within session average raw RT and TE for each animal was examined across the duration of the experiment and shown in Fig 5. Only NHP A showed a large fluctuation (> 200 ms) in RT across sessions. These fluctuations were not associated with the FUS with MB procedures. NHP N did show an increase in RT to the cue during days 150–178 but returned back to baseline. Similarly there was a trend of increased RT to the target for NHP N starting on day 77 in which the RT remained elevated for the remainder of the experiment. Average raw TE was more consistent for each NHP, fluctuating less than 5 mm for NHP N and Ob. NHP A showed a sharp decrease in TE to the correct target over the first 30 days then remained at the lower TE value the remainder of the experiment.

**Fig 6:**
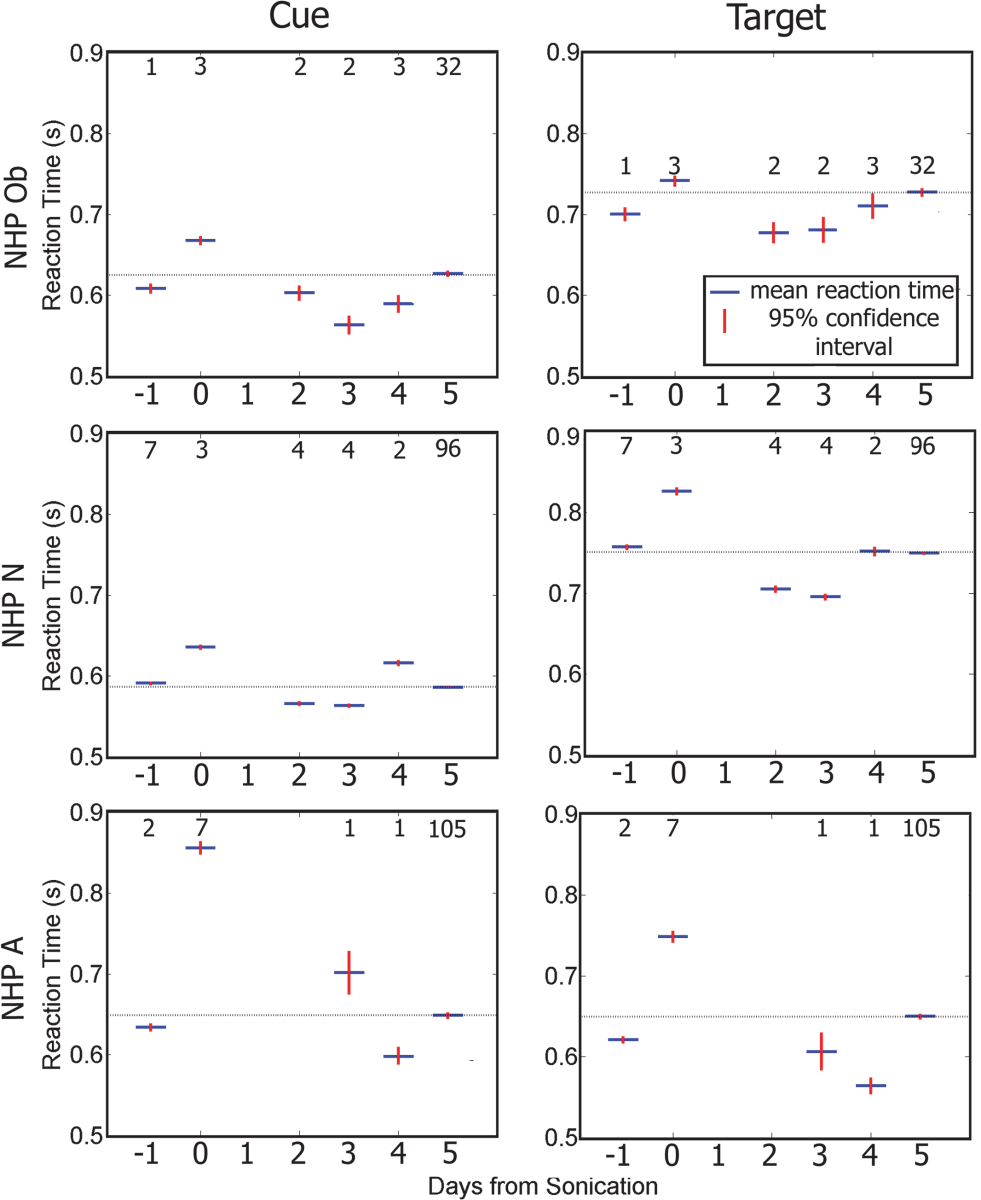
Average reaction time to initial cue (left column), and to the correct target (right column). Blue indicates group average while red is the 95% confidence interval for the mean. For all NHP there is a significant increase in reaction time on day 0 compared to the rest of the days (p < 0.05). The numbers above each average indicate the n value for that group. The horizontal bar indicates baseline reaction time.

Fig 6 shows data groupd by day relative to the day of sonication. Average RT to the cue and target increased significantly (one-way ANOVA with Tukey’s HSD criterion p < 0.05) for all NHP on the day of the FUS with MB procedure compared with other days. Fig 6 shows that for NHP N and Ob there was a decrease in RT on days 2 and 3 after the FUS with MB procedure compared to the other days. A similar decrease was only observed on day 4 for NHP A. Within five days RT had returned to baseline for all NHP.

**Fig 7:**
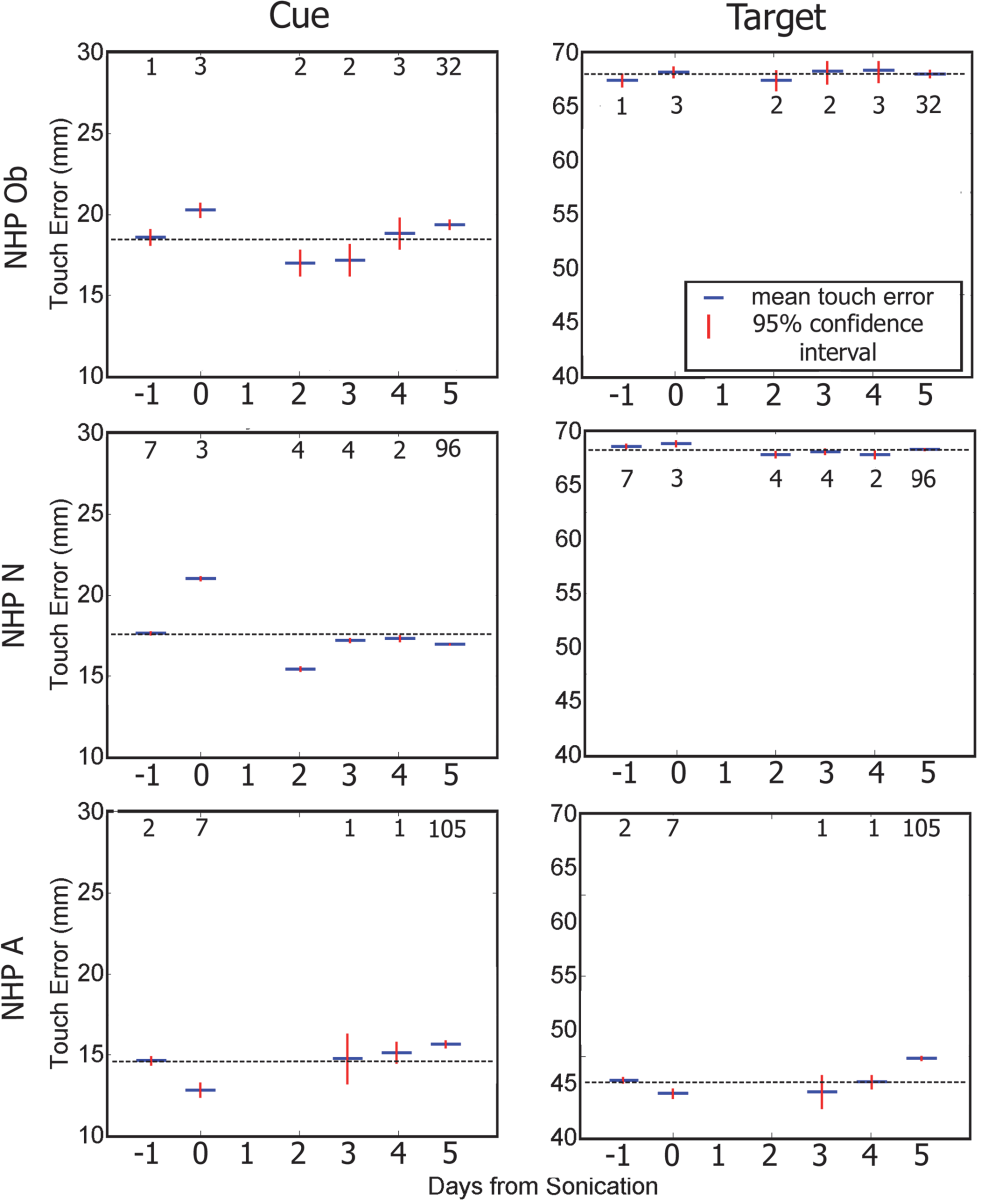
Average touch error to initial cue (left column), and to the correct target (right column). Blue indicates group average while red is the 95% confidence interval for the mean. There was a significant difference touch error to the cue on day 0 and the majority of the rest of the days (p < 0.05). There was a significant difference touch error to the correct on day 0 and some of the other days (p < 0.05). The numbers above each average indicate the n value for that group. The horizontal bar indicates baseline reaction time.

We assessed touch error (TE) as the distance from where the NHP touched the screen to the center of the cue or target stimuli. Reaching errors are shown in Fig 7. The average TE to the cue increased significantly for NHP Ob and N but decreased for NHP A on the day of the FUS with MB procedure compared to the other days (one-way ANOVA with Tukey’s HSD criterion p < 0.05). As shown in Fig 7, the TE to the correct target had less variance than the TE to the cue for all NHP. There was no significant change in TE to the correct target on day 0 compared with the rest of the days for NHP Ob. NHP N and A showed multiple days with significantly different TE to the correct target from that of day 0.

**Fig 8:**
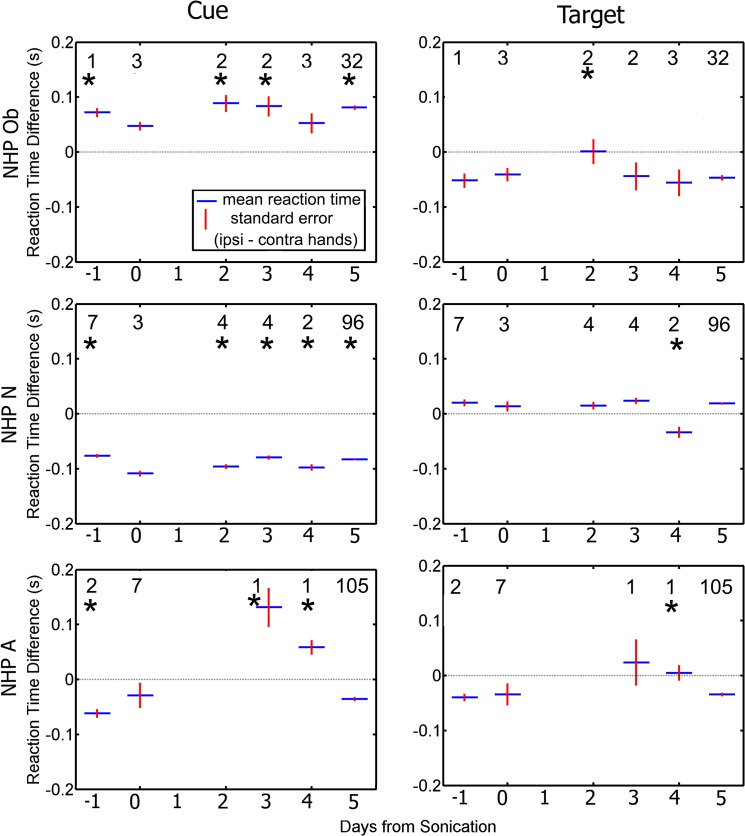
Difference in average reaction time between the ipsilateral and contralateral hands as a function of day relative to the day of the FUS procedure. Responses to the cue are plotted in the left column. Responses to the target are plotted in the right column. Blue indicates group average (average ipsilateral hand reaction time—average contralateral hand reaction time) while red is the standard error of the mean. The numbers indicate the n value for that group. Asterisks above each average indicate a significant difference between the difference in reaction time on day 0 compared to the rest of the days (p < 0.05).

Each FUS procedure was performed on only one hemisphere at a time. It might be expected that sonication would have stronger effects on reaches made with the hand contralateral to the sonicated hemisphere. We quantified this by separating responses made with the hand contralateral or ipsilateral to the treated hemisphere. We used the ipsi-contra difference because overall RT and TE can vary from day to day. Fig 8 shows the average ipsi-contra RT difference for each day. When initially reaching for the cue, all NHP showed a significant hand bias on most non-sonicated days (student t-test, p < 0.05), but the bias was not consistent across animals; Ob was faster with the contra hand, N was faster with the ipsi hand and A showed different biases on different days. The biases for all NHP were smaller and less likely to be significant when reaching for the target. On sonication days (day 0), none of the NHP showed significant hand biases when reaching for either the cue or target.

**Fig 9:**
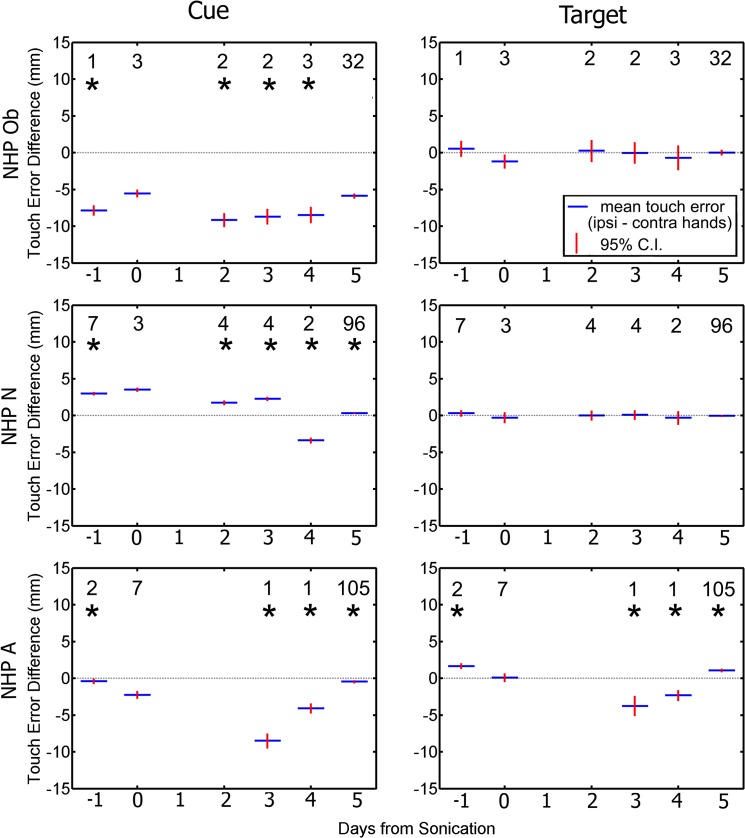
Difference in average touch error between ipsilateral and contralateral hands as a function of day relative to the day of the FUS procedure. Responses to the cue are plotted in the left column. Responses to the target are plotted in the right column. Blue indicates group average (average ipsilateral hand touch error—average contralateral hand touch error) while red is the standard error of the mean. The numbers indicate the n value for that group. Asterisks above each average indicate a significant difference between the difference in reaction time on day 0 compared to the rest of the days (p < 0.05).

Fig 9 shows hand bias for touch accuracy as the difference in average TE between ipsilateral and contralateral. Similar to the RT results, all NHP show significant hand biases (student t-test, p < 0.05) when reaching to the cue on non-sonicated days, but no significant differences on the day of sonication. When reaching for the target, no NHP showed a significant hand bias on the day of sonication. Only NHP A showed significant differences on non-sonicated days.

Considering the RT and accuracy data together, sonication tended to reduce the significance of the animal’s pre-existing hand biase. However, because these biases were idiosyncratic, sonication did not systematically make reaches with the contralateral hand slower or less accurate.

**Fig 10:**
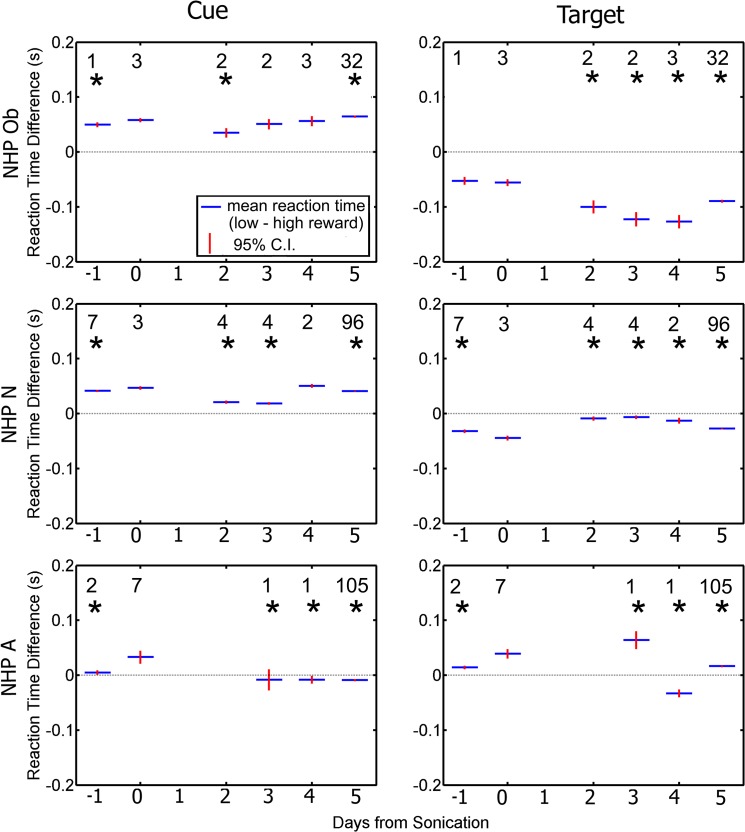
Difference in average reaction time between low and high reward as a function of day relative to the day of the FUS. Responses to the cue are plotted in the left column. Responses to the target are plotted in the right column. Blue indicates group average (average low reward reaction time—average high reward reaction time) while red is the standard error of the mean. The numbers indicate the n value for that group. Asterisks above each average indicate a significant difference between the difference in reaction time on day 0 compared to the rest of the days (p < 0.05).

Differences in RTs to high and low reward stimuli can be an index of motivation. Fig 10 shows the difference in RT between the low and high reward to the initial cue and correct target. On non-sonicated days, NHP Ob and N were faster in reaching for the initial cue and slower in reaching for the target when the reward was high. NHP A showed the opposite pattern. On sonicated days, reward magnitude had no effect on reaction time for either the cue or target.

**Fig 11:**
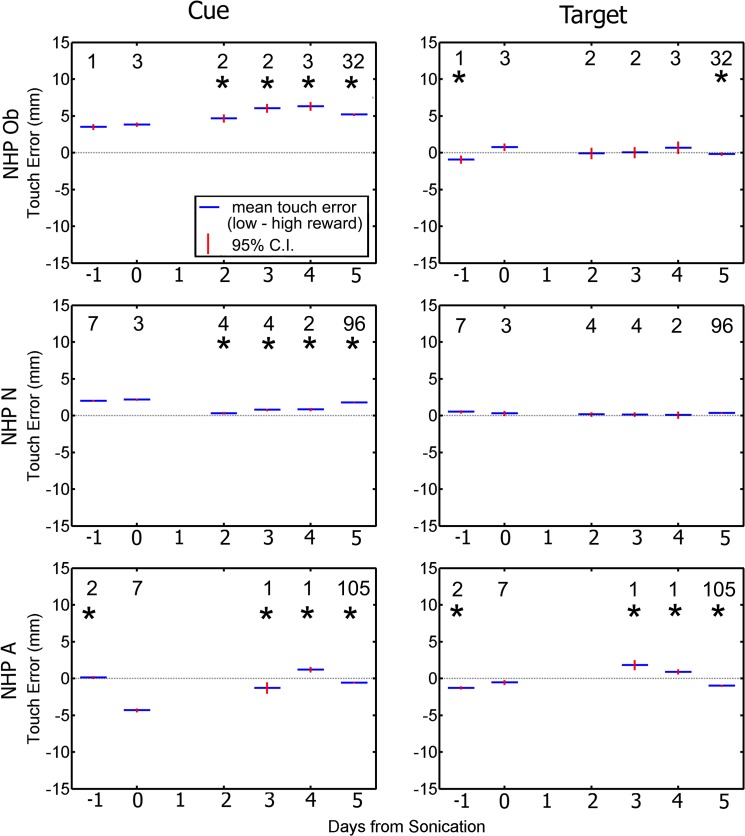
Difference in average touch error between low and high reward as a function of day relative to the day of the FUS. Responses to the cue are plotted in the left column. Responses to the target are plotted in the right column. Blue indicates group average (average low reward touch error—average high reward touch error) while red is the standard error of the mean. The numbers indicate the n value for that group. Asterisks above each average indicate a significant difference between the difference in reaction time on day 0 compared to the rest of the days (p < 0.05).

The difference between the low and high reward on TE was also investigated. Fig 11 shows the average difference in TE between the low and high reward for both the initial cue and the correct target. On non-sonicated days, NHP Ob and N tended to be less accurate in reaching for the cue on low-reward trials (as well as being slower, as shown in Fig 10). When reaching for the target, Ob and N rarely showed any accuracy difference between low and high reward trials. NHP A showed mixed results when reaching for either the cue or target. On sonicated days, none of the NHP showed any difference in accuracy between high and low reward trials.

Considering the RT and accuracy data together, all NHP showed significant differences between high and low rewards on non-sonication days. These differences could be attributed to the motivational significance of reward size, especially NHP Ob and N’s tendency to be faster and more accurate on high reward trials when reaching for the cue. The lack of any significant difference in RT or accuracy on sonicated days suggest that sonication may have slightly dampened the NHP’s motivation to reach faster and more accurately for large rewards.

The previous results raise the question of whether sonication affects the NHP’s cognitive abilities. Decision-making can be assessed by performance accuracy, i.e. frequency of selecting the correct target. Overall each animal exhibited > 76% accuracy in selecting the target indicated by the dot direction which is significantly over chance. This accuracy did not significantly change on days when the FUS with MB procedure occurred (student t-test, p > 0.05).

**Fig 12:**
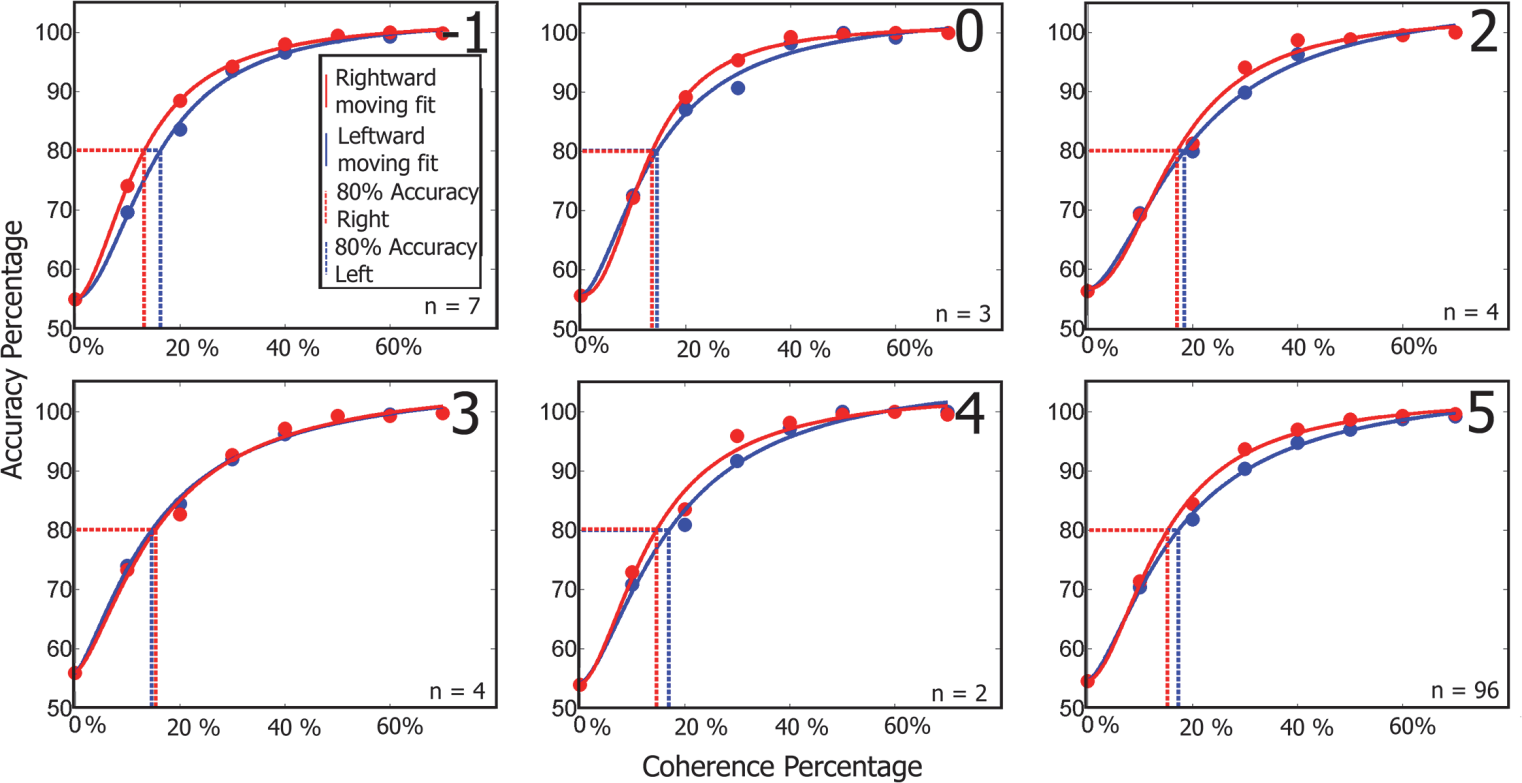
Naka-Rushton model fits of accuracy against coherence for the RDM task completed by NHP N. The red and blue circles indicate the average percent correct for each coherence level for the right and leftward moving dots respectively. The numbers in the top left corners correspond to days relative to the FUS with MB procedure. Other NHP coherence plots follow similar trends of no large variation in response to the FUS with MB procedure.

A more sensitive measure of decision-making is the coherence threshold for identifying direction of motion. Coherence threshold is the percentage of coherently moving dots for which the subject correctly judged motion direction on 80% of the trials. The average coherence thresholds for all NHP were at or below 31%. Fig 12 plots percent correct direction discrimination as a function of motion coherence for NHP N. The solid curves are the Naka-Rushton curve fits to the raw data. Results for NHP Ob and A were similar. NHP N exhibited the lowest average coherence thresholds across groups for both right and leftward moving dots at 15% and 17% respectively. Coherence thresholds for each group are shown in Table 4. Thresholds were not significantly elevated on the day of sonication (day 0), if anything, they were lower, indicating that sonication did not impair (and may have improved) the NHPs motion perception or decision-making.

**Table 4:** Dot coherence percentages for 80% accuracy.

Day	-1	0	2	3	4	5
A Right	19%	13%	N/A	24%	15%	22%
A Left	14%	22%	N/A	33%	23%	26%
N Right	13%	14%	17%	15%	15%	15%
N Left	16%	15%	19%	15%	17%	17%
Ob Right	22%	30%	66%	N/A	31%	41%
Ob Left	24%	19%	34%	30%	27%	34%

Table is divided into days relative to the FUS with MB procedure, animal and the direction of the moving dots.

The behavioral data recorded on the days when the hyperintense voxels were observed on the T2-weighted MRI scans did not significantly vary from data acquired when there were no hyperintense voxels detected (student’s t-test, p < 0.05). Average RT and TE were not significantly different from the data acquired on other days when the FUS with MB procedure occurred. The difference in RT between the contralateral and ipsilateral hands also did not significantly vary from the other days when the procedure had occurred.

## Discussion

A major hurdle for developing therapies prior to clinical trials is determining the safety of the procedure. Previous studies have shown that short term applications of FUS with MB can safely open the BBB in multiple *in vivo* models such as mice and NHP. Here we furthered verified the safety for long term applications of FUS with MB BBB opening in NHP through vital sign monitoring, MRI analysis and behavioral testing. Our combined results show that FUS-mediated BBB opening in the basal ganglia does not have long term effects on the general physiology of the NHP, the structure of the targeted brain regions nor on decision and motor function.

### Safety

As this technique moves closer to clinical testing, the safety of the procedure must be thoroughly investigated. During surgical procedures, heart rate, respiration rate, and blood pressure all increase with stimulation (cutting, drilling) even after the NHP has been at a deep steady state of anesthesia for prolonged periods [34]. The vital signs monitored during the FUS with MB procedure did not exhibit any significant variations outside of normal cardiovascular or pulmonary function. Fig 2 shows inter-NHP but no intra-NHP variations in heart rate, blood pressure, respiratory rate, CO_2_ emissions and SPO_2_ levels. An initial drop of the heart rate occurred after induction of anesthesia and can be attributed to the effects of isoflurane (Fig 2).

Agreeing with previous long term studies on mice, we did not observe any gross physiological changes in weight, food and water consumption, activity levels, mobility or emotional state with the NHP over the course of repeated FUS with MB procedures [18]. The contrast enhanced T1-weighted MRI scans obtained at least 5 days after the FUS with MB procedure at the middle and end of the experiment did not indicate increased permeability in the targeted regions. These scans show that repeated BBB opening via the FUS with MB applications does not permanently increase the permeability of the targeted area.

T2-weighted MRI and SWI sequences were used to determine possible damage at the BBB opening region. Previous work suggests that the MB size and acoustic pressure are the critical parameters in dictating both the opening size and safety [35],[36]. Only NHP N and A exhibited hyperintense spots in the T2-weighted MRI sequences for one and three applications of FUS with MB, respectively. The hyperintense spots on the T2-weighted scans suggest possible, blood, or edema [37]. There was no hypointense signal in the same area for the SWI scans, which eliminates the possibility of hemorrhage [38]. To rule out permanent lesions T2-weighted and SWI sequences were acquired a week after the initial detection and revealed no hyperintense or hypointense voxels in the region where they were previously observed (Fig 4). Thus, the hyperintense spots could have been caused by edema which was cleared over the course of a week. The acoustic pressures used for these cases were 300kPa for NHP A and 400kPa for NHP N with 4–5μm MB. Previous pressures of 440–700 kPa with Definity MB were shown to cause hypointense spots in T2*-weighted imaging as well as hemorrhaging in the thalamus region after histological investigation [21]. In the current study, the NHP were not euthanized as they were already selected to be used in future experiments and therefore histology was not available. This is a limitation to the scope of this study as the histology might have revealed blood cell extravasation, neuronal death or an immune response in the areas of repeated BBB opening. From previous studies where histology was conducted after short term application of the FUS with MB procedure on NHP we could expect some petechaie and possible damaged capillaries [21]. No significant variations in RT or TE on days when the hyperintense voxels were detected compared to the days with no hyperintense voxels. Thus the presence of possible edema did not have an effect on the behavioral results for that given day. Over the course of all FUS with MB procedures there was no change in the NHP ability to perform daily functions or a change in their disposition. With the parameters and targets selected within this study we believe repeated FUS with MB procedures can be safely applied long term.

As mentioned previously the parameters utilized for the FUS with MB procedures within this study were originally derived from previous studies in our lab [11],[20]. We found that although the NHP are within the same relative size/age group, each NHP has FUS parameters that are optimal for them. NHP A and O were smaller subjects (5–6kg) and substantial BBB opening could be achieved with lower pressures (300 kPa), while NHP N and Ob (8–9kg) were physically larger and required higher pressures (400 kPa) to achieve similar opening sizes. These selected pressures also allowed for safe BBB opening with the majority of the experiments conducted in this study. This supports the notion that while there are general guidelines for parameters ensuring safe BBB opening for a specific species, optimized parameters should be identified for individual subjects. This will be important in the future when the FUS with MB procedure makes the step into the clinic with patients.

### Behavioral task

The regions of the basal ganglia targeted in these experiments are involved in decision-making and motor control. Gold and colleagues have shown that neurons in the caudate nucleus of NHP signal decision variables during a random-dot motion task similar to the one used in this study [39]. Hikosaka and colleagues have documented the involvement of the caudate in reward-based reaction-time differences [26],[27]. Pilot experiments in our lab undertaken in preparation for the current study also provided evidence that targeting the basal ganglia with FUS with MB can have profound behavioral effects. In one NHP (M, adult male rhesus), unilateral delivery of FUS with MB to the caudate resulted in hemispatial neglect contralateral to the treated hemisphere that lasted for roughly 24 hours. This was likely due to excessive FUS pressure. In NHP N and Ob, FUS with MB targeting the caudate and coupled with IV delivery of the D2 antagonist domperidone also resulted in contralateral neglect, oculogyric crisis, and circling to the contralateral side. These symptoms lasted several hours. These experiments were not repeated due to problems with systemic injection of domperidone. For the results reported from the experiments in this study, no NHP responded to the FUS with MB procedures with physical deficits as discussed above.

The RDM + RMB task was well suited for determining if there were any effects of the FUS with MB procedure on either the motor signal pathway or the decision making pathways associated with the basal ganglia[22],[29]. Movement commands initiated in the motor cortex pass through the basal ganglia and the cerebellum before being sent to the spinal cord [29]. The dorsal parts of the caudate and putamen are associated with sensorimotor function while the ventral parts are associated with limbic functions [40]. Thus, if the FUS with MB procedure disrupted the pathways in the basal ganglia regions, motor and decision making deficits should be observable [41]. It is of interest that there was a significant increase in the RT to both the initial cue and the correct target on the day of the FUS with MB procedure for all NHP, but this returned to baseline within five days. Similarly the average TE to the initial cue was elevated for NHP N and Ob and decreased for NHP A, which also returned back to baseline within 5 days. Behavioral testing that occurred on the same day as the FUS with MB procedure was done several hours after the procedure, and therefore after a period of about an hour of anesthesia. However, it is unlikely that the isoflurane had an effect on behavioral responses. It has previously been shown that isoflurane has the fastest recovery time of anesthetic drugs for NHP with a recovery time of 20 minutes even for high doses (3–4%) [42]. We applied lower doses of isoflurane (2% max when placing the NHP into the stereotax) and decreased the dosage down to 0.5% during the final five minutes of the procedure minimizing their total exposure to anesthesia.

Examining the RT difference between hands was a useful indicator if the FUS with MB procedure had disrupted the motor processing pathways in the basal ganglia as only one hemisphere was targeted during each procedure. Similar to humans, NHP have a preferred hand for most tasks, and thus have faster RT for that hand [43],[44]. This hand preference can be seen on the baseline days (-1, 5+) for the RT to the cue for all NHP in Fig 8. If the FUS with MB procedure had affected the basal ganglia only in the targeted hemisphere, the effects should have been observed in the contralateral hand, thus changing the difference in RT between the two hands. As the average difference RT between the ipsilateral and contralateral hand to the initial cue for NHP N and A is below 0 indicating his ipsilateral hand is dominant for both the baseline days and days where the FUS with MB procedure occurred. Similarly average difference in RT between hands for NHP Ob is above 0, indicating his contralateral hand is dominant. Interestingly this dominance switches when responding to the correct target for both NHP Ob and N. This inversion in dominance to the correct cue and correct target was not observed in NHP A. Regardless, hand dominance is not affected by the FUS with MB procedure for any of the NHP. Our results do not indicate that the FUS with MB procedures have an effect on handedness nor specifically on the RT of the contralateral hand for two of the three NHP.

As expected with the RMB portion of the task, most of the NHP responded with faster RT to the high reward cue, than the low reward cue for most of the days as the average diference between the low and high reward is above 0 (RT to cue seen in Fig 8). These results agree with previous studies where NHP made saccadic eye movements to complete an RMB task [26]. In that study, NHP had faster saccades to the high reward, while slower saccades to the low reward. The bias in responding faster to the high reward is reversed for RT to the correct target as the average difference in RT between the low and high reward is below 0 indicating a faster response to the low reward. This could be attributed to a speed accuracy tradeoff, as the higher reward was more salient and thus the NHP took additional time to select the correct target [45]. The responses from NHP A were more varied across days and did not show the bias that was observed in both NHP N and Ob. The bias seen with NHP N and Ob was not affected by the FUS with MB procedures. Overall the FUS with MB procedures did not have an effect on the reward bias for the two NHP that followed the paradigm originally.

Touch error was an important factor for determining if the FUS with MB procedure had an effect on the basal ganglia. If the average distance between the target and the point where the NHP touched the screen increased or became more erratic between separate days to the point of significant variation, it could indicate that the FUS BBB opening procedure had an effect on the voluntary motor control pathway [42]. As with Parkinson’s, the disruption of the motor pathway can lead to undershooting when reaching for a target [46]. As seen in Fig 7 most NHP showed a significant difference between day 0 and the other days for the initial cue. There were fewer days with a significant difference in TE to the correct target between day 0 and the other days. This could have been caused from the prior position of the NHP hand before selecting the correct target as it would be in the relative same position for each trial having just selected the initial cue. There would be more variation in TE to the initial cue as the NHP could have its hand resting in various positions before reaching to the initial cue, increasing the variability of the TE. NHP N did not exhibit any significant variation of the TE to the target between day 0 and the other days over the duration of the behavioral recordings independent of hand or reward magnitude. NHP Ob only showed two days where there was a significant difference in TE to the correct target between day 0 and the rest of the days with respect to reward magnitude, but similar to NHP N did not show any significant variation with respect to the difference between hands. This indicates the FUS with MB procedure did not have an effect on touch error to the target.

The RDM component of the task tested whether the FUS with MB procedure was having an effect on the decision making pathways associated with the basal ganglia [40]. The coherence threshold in Fig 10 does not vary more than 4% across each group. The variance of the detection threshold between days and the individual daily threshold for detection was low and consistent with previous investigations using the RDM task for the majority of the days [47],[48]. NHP Ob exhibited the largest variation between baseline coherence threshold and the day of the FUS with MB procedure with a 10% variation, while NHP A and N exhibited less than 8% variation. This percentage is comparable to variation between non-FUS with MB procedure days and does not indicate the FUS with MB procedure had an effect on the decision making pathways in the basal ganglia of the NHP subjects.

## Conclusion

As FUS mediated BBB opening moves closer towards clinical feasibility, the safety of repeated FUS BBB opening procedures must be characterized in the NHP model. Here we showed that repeated BBB opening at the caudate and putamen regions in NHP can be achieved safely without hemorrhage or permanent edema, and to not cause a permanent effect on RT and decision making responses with the applied FUS parameters. Our findings support that FUS is a promising technique for clinical applications as it is the only non-invasive procedure that can be used to chronically and accurately open the BBB safely in both cortical and subcortical regions of the brain without causing damage to the structure or neurological pathways within it.

## Supporting Information

S1 Checklist The required ARRIVE Guidelines Checklist associated with the *in vivo* experiments conducted in this study. (DOCX)
