# Correction: Long-Term Safety of Repeated Blood-Brain Barrier Opening via Focused Ultrasound with Microbubbles in Non-Human Primates Performing a Cognitive Task

**DOI:** 10.1371/journal.pone.0130860

**Published:** 2015-06-18

**Authors:** Matthew E. Downs, Amanda Buch, Carlos Sierra, Maria Eleni Karakatsani, Tobias Teichert, Shangshang Chen, Elisa E. Konofagou, Vincent P. Ferrera

Dr. Tobias Teichert should be included in the author byline. He should be listed as the fifth author, and his affiliation is 4: Psychiatry Department at the University of Pittsburgh, Pittsburgh, Pennsylvania, United States of America. The contributions of this author are as follows: Conceived and designed the experiments, performed the experiments.

The correct citation is: Downs ME, Buch A, Sierra C, Karakatsani ME, Teichert T, Chen S, et al. (2015) Long-Term Safety of Repeated Blood-Brain Barrier Opening via Focused Ultrasound with Microbubbles in Non-Human Primates Performing a Cognitive Task. PLoS ONE 10(5): e0125911. doi:10.1371/journal.pone.0125911
