# Supplementary material for: Long-Term Safety of Repeated Blood-Brain Barrier Opening via Focused Ultrasound with Microbubbles in Non-Human Primates Performing a Cognitive Task
Source: PLoS One. 2015 May 6;10(5):e0125911. doi: 10.1371/journal.pone.0125911 (PMC4422704; doi:10.1371/journal.pone.0125911)
Supplement: S1 Checklist — (DOCX) [file pone.0125911.s001.docx]

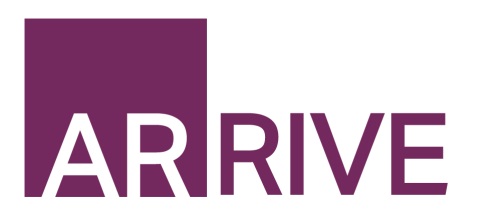


The ARRIVE Guidelines Checklist

Animal Research: Reporting In Vivo Experiments

Carol Kilkenny^1^, William J Browne^2^, Innes C Cuthill^3^, Michael Emerson^4^ and Douglas G Altman^5^

*^1^The National Centre for the Replacement, Refinement and Reduction of Animals in Research, London, UK, ^2^School of Veterinary Science, University of Bristol, Bristol, UK, ^3^School of Biological Sciences, University of Bristol, Bristol, UK, ^4^National Heart and Lung Institute, Imperial College London, UK, ^5^Centre for Statistics in Medicine, University of Oxford, Oxford, UK.*

|  | | ITEM | | RECOMMENDATION | Section/ Paragraph |
| --- | --- | --- | --- | --- | --- |
| 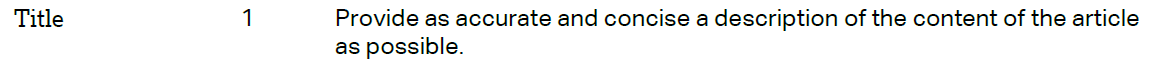 | | | | Long-Term Safety of Repeated Blood-Brain Barrier Opening via Focused Ultrasound with Microbubbles in Non-Human Primates Performing a Cognitive Task |  |
| 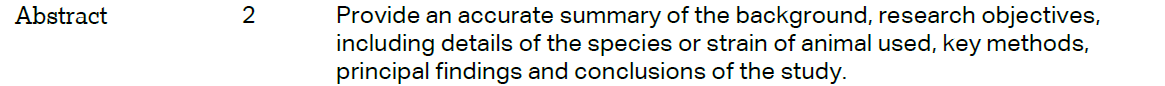 | | BACKGROUND AND PURPOSE: Focused Ultrasound (FUS) coupled with intravenous administration of microbubbles (MB) is a non-invasive technique that has been shown to reliably open (increase the permeability of) the blood-brain barrier (BBB) in multiple *in vivo* models including non-human primates (NHP). Our aim here was to investigate the long term safety effects of this procedure on NHP.  EXPERIMENTAL APPROACH: Four NHP had the FUS with MB procedure applied to them over a duration of 4-20 months targeting the basal ganglia (500kHz, 200-400 kPa, 4-5µm MB, 2 minute sonication). Safety of the procedure was verified with MRI (T1-weighted, T2-weighted, Susceptibility Weighted Imaging; n = 4 NHP) and through behavioral testing (reward magnitude bias + random dot motion; n = 3 NHP).  KEY RESULTS: Applying the FUS with MB procedure over a duration of 4-20 months with the parameters stated above did not have elicit any detectable negative effects. The MRI scans showed no long term damage. Results from the behavioral tasks showed that the procedure did not have an effect on the neural pathways within the basal ganglia.  CONCLUSIONS AND IMPLICATIONS: These results show that the FUS with MB procedure is safe for long term repeated application at the targeted location location. This is an important finding as it moves this procedure closer towards clinical testing in human subjects. | | |  |
| INTRODUCTION | | | |  |  |
| 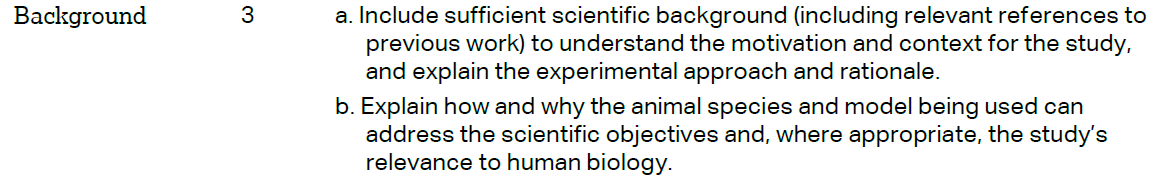 | | 1. The blood brain barrier (BBB) is a highly selective biological system that maintains brain homeostasis [1]. As with many efficient systems, it has negative consequences, namely preventing 99% of currently available small molecules (> 400 Da) and all large molecule drugs from crossing the BBB [2]. This hinders clinical treatments of neurological diseases and disorders, as well as development of novel drugs to treat the aforementioned diseases [3,4]. Current techniques for drug delivery through the BBB are either invasive and localized or non-invasive with regionally nonspecific delivery [5,6,7]. Our group and others have shown over the past decade that focused ultrasound with microbubbles is an effective technique to open the BBB for multiple *in vivo* animal models [8,9,10,11]. The introduction of MB coupled with lower-pressure FUS has been shown not to generate tissue damage or neurological deficits in mice [12,13]. Our group and others have shown that for specific parameters the FUS with MB procedure can be safe for non-human primates (NHP) [14,15]. The primary method of evaluating potential damage from FUS BBB opening in NHP is MRI, specifically T2-weighted and Susceptibility-Weighted Imaging (SWI) scans [14]. There has been one study utilizing histological evaluation of the FUS BBB opening procedure in NHP over a time frame of 2-26 weeks [15]. While MRI and histological evaluation are useful for detecting cellular damage from the procedure, neither method can detect if the FUS with MB procedure has an effect on neurological function. A previous study reported the effects of several weeks of FUS with MB application on the thalamus (lateral geniculate nucleus) in the NHP model using a visual acuity task [15]. To date there has not been any study conducted on the neurological effects on motor and cognitive processing of repeated (> 13 months) FUS with MB procedures with BBB opening in NHP. 2. NHP were selected for this procedure as they have the closest physiology to humans of the *in vivo*  models and are regularly used to test the safety of procedures or drugs before human clinical testing | | |  |
| 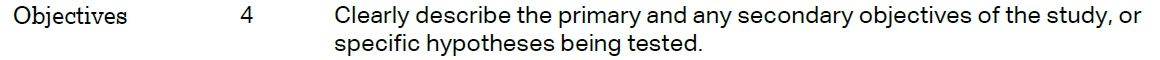 | | The objectives of this study were to determine the long term effects of repeated FUS with MB procedures on the basal ganglia of NHP. The safety of the procedures were verified with MRI analysis (for potential structural tissue damage) and with behavioral testing (to determine if the procedure was having an effect on the neural pathways within the basal ganglia) over a time span of 4-20 months. | | |  |
| METHODS | |  | | |  |
| 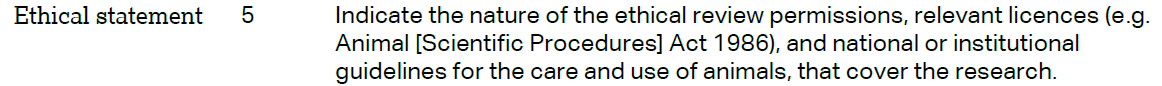 | | All NHP procedures described herein were approved by the Institutional Animal Care and Use Committees of Columbia University and the New York State Psychiatric Institute (protocol number AC-AAAF2715). | | |  |
| 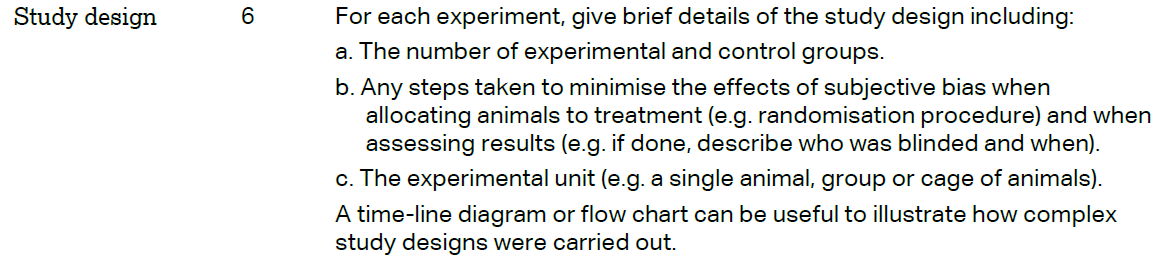 | | 1. Each animal acts as its own control group as we are only applying the FUS with MB procedure to one hemisphere of the brain during each experiment. The untreated hemisphere acts as an in-animal control. For the initial study verifying the safety of the FUS with MB procedure only using MRI scans four NHP were used (n = 4). The later experiments using both MRI scans and behavioral testing, three NHP were used (n = 3). Each NHP had the FUS with MB procedure applied to them at minimum 4 times and at maximum 27 times. 2. No randomization was used. It was clear to those conducting the experiment which hemisphere the FUS with MB procedure was being applied to for both the initial and later experiments. The three NHP chosen for the behavioral experiment were able to become proficient in the behavioral task. The last NHP was not able to become proficient in the task before behavioral recordings began and was thus excluded from that portion of the experiment. 3. Four NHP for the initial safety assessment of the FUS with MB procedure (n = 4), three NHP for the behavioral testing portion (n = 3). | | |  |
| 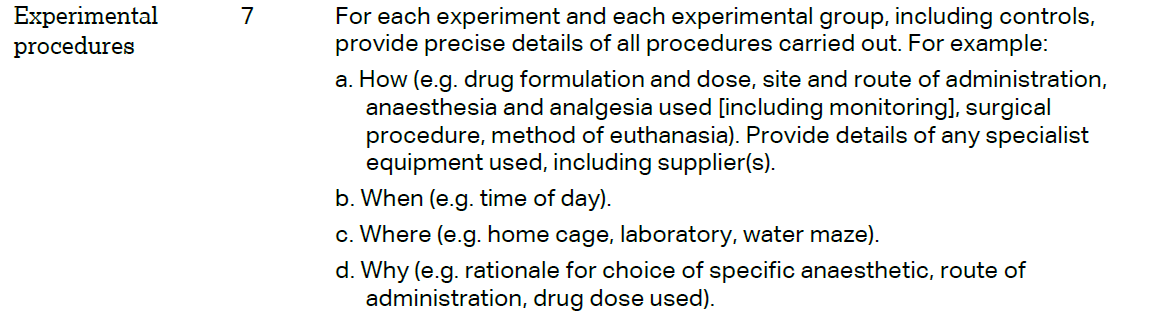 | | 1. For the FUS with MB procedure, NHP were initially sedated with ketamine (10-12 mg/kg) and given a dose of atropine (0.04mg/kg), both delivered IM. An endotracheal catheter was inserted, after which the NHP were anesthetized with isoflurane (1-2%) mixed with O_2_ (2 L/min) for the duration of the procedure. The NHP were sedated for all applications of the FUS with MB procedures. A heated water blanket was used to maintain body temperature during the FUS with MB procedure. Heart rate (EKG), blood oxygenation (SpO_2_), end-tidal CO_2_ expiration, respiratory rate, and non-invasive blood pressure were recorded during the FUS with MB procedure.  Anesthesia for the acquisition of MRI scans was the same as for the FUS with MB procedure. Monitoring of the anesthesia for the MRI scans was done with a respiration belt and EKG.   For the behavioral testing no drugs were administered to the NHP as it was not a painful or distressing procedure. 2. The FUS with MB procedures either occurred around 9:00 AM or 3:30 PM lasting approximately 1-1.5 hours. MRI scans occurred either at 5:00 or 7:00 PM lasting approximately 2 hours. The behavioral testing occurred between the hours of 9:00 AM – 5:00 PM and lasted approximately 2-4 hours. 3. The FUS with MB procedures occurred in the surgery suite of the same floor as the animal husbandry rooms. The MRI scans occurred in the Hatch Center for MRI research. For the behavioral testing the NHP were placed in specially designed NHP chairs which allowed free movement of their head and arms to complete the behavioral task. The behavioral testing occurred within specialized soundproof recording rigs to minimize outside interference with the experiment. 4. The aesthetic drugs chosen for the FUS with MB and MRI procedures are those commonly used for surgical procedures and are safe and effective for the NHP. | | |  |
| 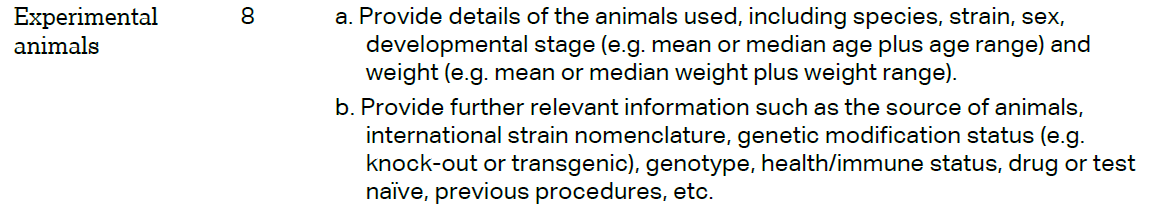 | | 1. Adult male macaques (n=4) were used in all experiments (ages 8-23 years, weights 5-9 kg); one Macaca fascicularis and three Macaca mulatta. Average age = 12.5 ± 5.9 years, average weight = 8.1 ± 1.3 kg 2. NHP were acquired at different times from different vendors. The vendors are as listed: Worldwide Primates Inc., Laboratory Animals Breeders & Services, and Charles River Laboratories. The animals were not genetically modified. They have all tested negative for herpes B and tuberculosis as well as other viral, bacterial or parasitic pathogens during their biannual tests. They were not previously used in any other experiments. | | |  |

| 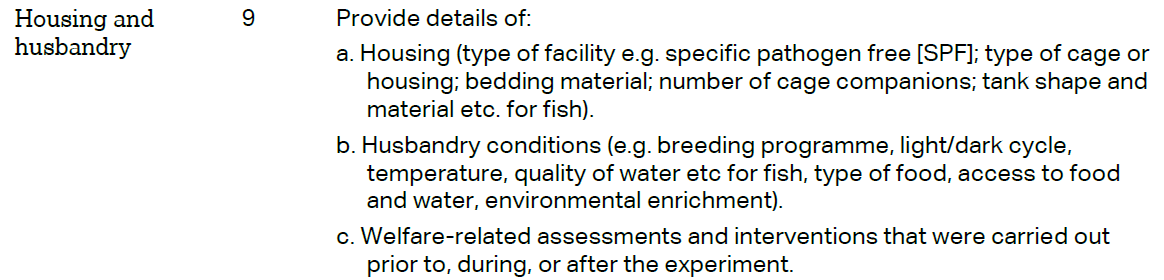 | 1. Housing consisted of the enhanced Environment Housing System from Primate Products Inc. Only two of the NHP were paired throughout the experiment. 2. Animals were on a 12 hour light/dark cycle (6 AM – 6 PM). Room temperatures were kept at 73°F±5°. Water was given freely on days when the behavioral task was not completed. On days when they completed the behavioral task water was given for correct responses and the NHP work until satiated. NHP were given a treat in the form of fresh or dried fruit and nuts after the completion of the behavioral task.Animals were provided High Protein Monkey Diet Jumbo (LabDiet 5047). All animals were allowed access at least once a week to the play cage and were always supplied with enrichment toys daily. 3. As mentioned in 7:a vital signs were closely monitored during all procedures where anaesthetics were used. When animals were removed from their housing to perform behavioral tasks their weight was recorded along with food, water and treat consumption. Biannually the animals were inspected by veterinary staff. During the experiment their weight as well as food and water consumption remained consistent and veterinary staff did not detect any physical abnormalities during biannual inspections. | |
| --- | --- | --- |
| 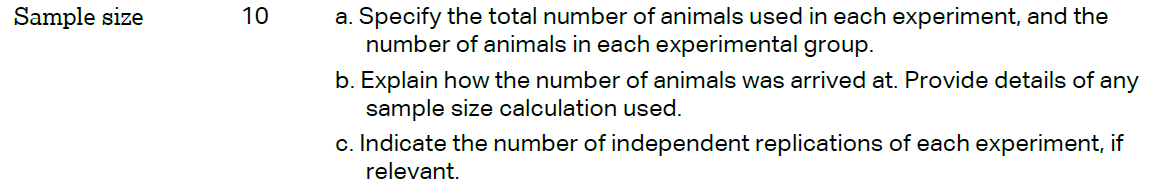 | 1. There were 4 NHP used for all the experiments. 4 NHP for the MRI safety verification study and 3 NHP for the behavioral task safety verification study. 2. The number of animals used in this study allows for a robust interpretation of the behavioral and safety data. It also accounted for variance in natural animal behavior to the task presented. 3. Each NHP completed the FUS with MB procedure at minimum 4 times. The NHP that were in the behavioral task safety verification study completed the task at minimum 48 times. | |
| 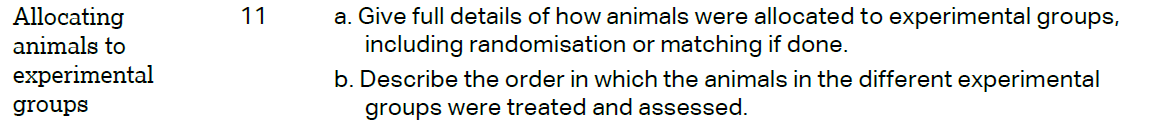 | 1. No random allocations occurred as there were only 4 NHP and they all underwent similar experimental procedures with both the FUS with MB procedure and behavioral testing. 2. The FUS with MB procedures were applied to each animal monthly for the duration of the study. The hemisphere targeted remained consistent for the MRI safety portion, and then for the behavioral testing portion. The behavioral testing was conducted at least twice a week for each animal in the behavioral task safety verification study. | |
| 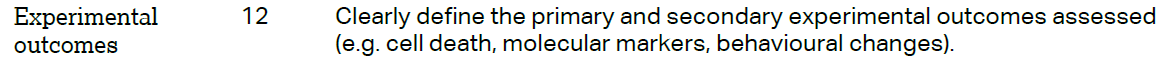 | Two primary outcomes were achieved with this study. The MRI scans verified that there were no long term effects of repeated FUS with MB procedures targeting the basal ganglia. Secondly, behavioral testing also concluded that the FUS with MB procedure targeting the basal ganglia did not have any effect on the neural pathways located there. | |
| 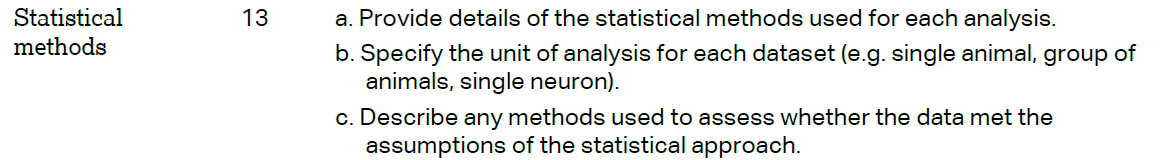 | 1. For the behavioral task data analysis was conducted as follows: The data were examined with two separate pipelines: The first was by dividing the data into groups depending on the acquisition of the data relative to the day of the FUS with MB procedure (-1, 0, 2, 3, 4 and 5+ days), and the second was by examining the data sequentially over the recording period. For the first method, days -1 and 5+ were considered to be a baseline since previous work has shown that the BBB openings created with the pressures being applied with this study close within three days [12]. This gives an additional two day buffer to ensure the BBB has completely closed. A one-way ANOVA with Tukey’s HSD criterion (p < 0.05) was used for analysis of the RT between groups to the cue and target. These groups were also divided into individual hand RT and analyzed with the student t-tests (p < 0.05 for significance) carried out between hands.  For the second pipeline the RT per day was divided by individual hands, and reward magnitude. Comparisons for RT between hands or reward bias for individual days were carried out with a student t-test (p < 0.05 for significance). A one-way ANOVA was used for detecting significant variance within each parameter across the duration of the experiment. Touch accuracy to the cue and target were also analyzed using the second pipeline. Touch accuracy was defined as the distance between where the NHP touches the screen and the center of the cue or target stimulus  Random dot motion accuracy was divided into groups using the same conditions as in the first pipeline. The data from each group was fitted on a psychometric curve (Naka-Rushton) and coherence thresholds were determined at 80% accuracy. 2. See above 3. All data sets met the requirements for utilization of the test employed in this study. | |
| RESULTS |  | |
| 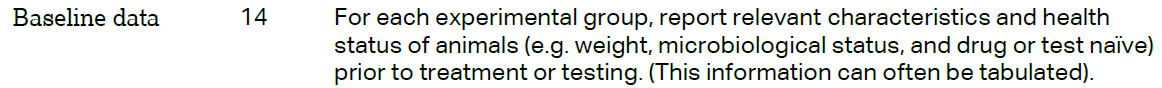 | As mentioned in Methods:9 the NHP weight as well as water and food intake were closely monitored throughout the duration of the experiment. There were no significant variations in the recorded values over the duration of the experiment. The vitals of each NHP were also monitored when the animals were sedated for the FUS with MB procedures and were not significantly different from previously reported values [16]. As also mentioned in the Methods:9 the NHP tested negative for Herpes B and tuberculosis as well as negative for other bacterial, viral and parasitic pathogens throughout the duration of the experiment. | |
| 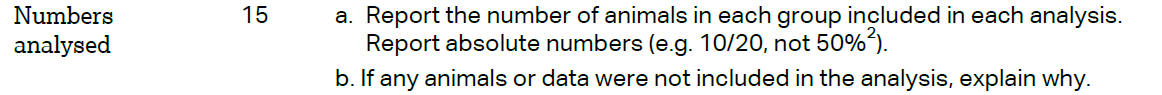 | Four NHP were analyzed for the MRI safety verification. Three NHP were analyzed for the behavioral testing safety verification. | |
| 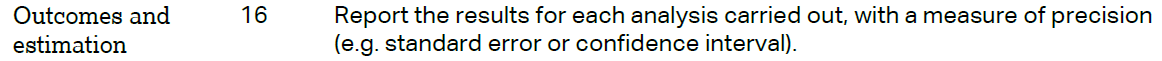 | 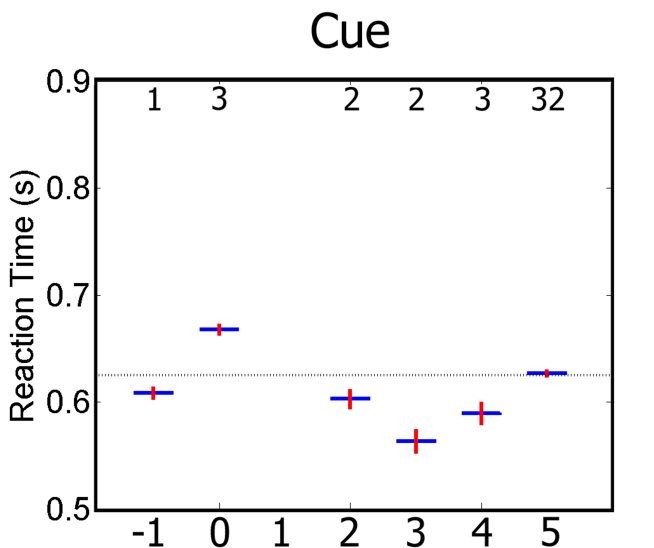In accordance with the ARRIVE guidelines (Kilkenny et al. 2010) we have reported all findings with precision and confidence along with n values in the full manuscript. As to report all outcomes of the data analysis here would be extensive. One figure is given as an example. The full analysis can be seen within the manuscript.  Here are the reaction times to the initial cue for one of the NHP broken down by groups. Groups are defined as days from the FUS with MB procedure, thus 0 is the day of the procedure. Horizontal blue lines indicate the average reaction time for the group. Vertical red lines indicate the 95% confidence interval of the mean. The dashed horizontal line indicates the baseline value determined by the average of group -1 and 5. The n values for each group (how many times behavioral recording occurred for each group) is indicated at the top of the figure. | |
| 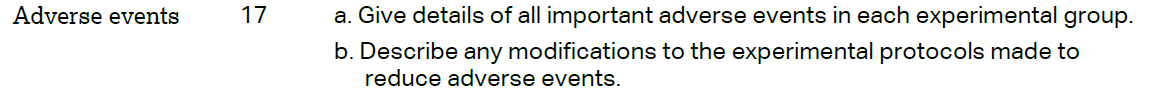 | There were no adverse effects of the procedures thus no modifications needed to occur. No NHP were sacrificed during these procedures. | |
| DISCUSSION |  | |
| 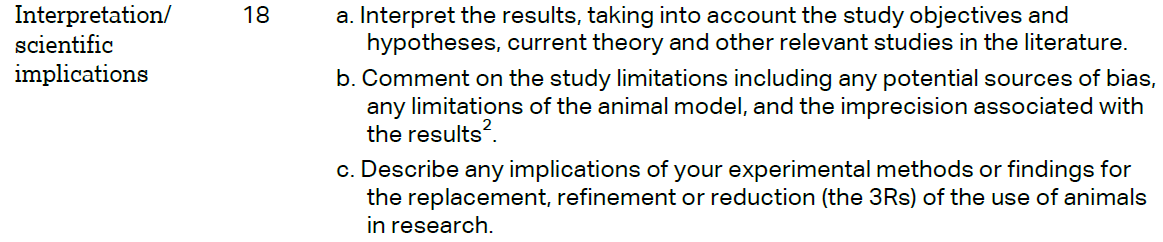 | A major hurdle for developing therapies prior to clinical trials is determining the safety of the procedure. Previous studies have shown that short term applications of FUS with MB can be a safe technique to open the BBB in multiple *in vivo* models such as mice and NHP. Here we furthered verified the safety for long term applications of FUS with MB BBB opening in NHP through vital sign monitoring, MRI analysis and behavioral testing. Our combined results show that FUS-mediated BBB opening does not have long term effects on the general physiology of the NHP, the structure of the targeted brain regions nor on decision and motor function in the putamen region of the basal ganglia. Our findings support that FUS is a promising technique for clinical applications as it is the only non-invasive procedure that can be used to chronically and accurately open the BBB safely in both cortical and subcortical regions of the brain without causing damage to the structure or neurological pathways within it. | |
| 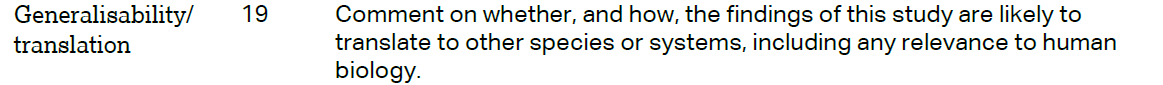 | Establishing the safety of the long term repeated application of the FUS with MB procedure in NHP is vital to move this technique closer to human clinical trials. A major end goal with this procedure is to treat humans with neurological diseases and disorders that cannot be currently treated in a non-invasive method. | |
| 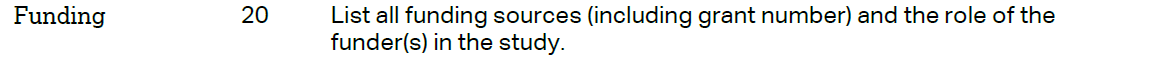 | | National Institute of Health R01AG038961 and R01EB009041; Wallace H. Coulter Foundation; FUS Foundation; Kavli Foundation; the Alfonso Martin Escuerdo Foundation  The funders had no role in study design, data collection and analysis, decision to publish, or preparation of the manuscript. |


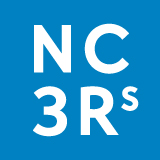

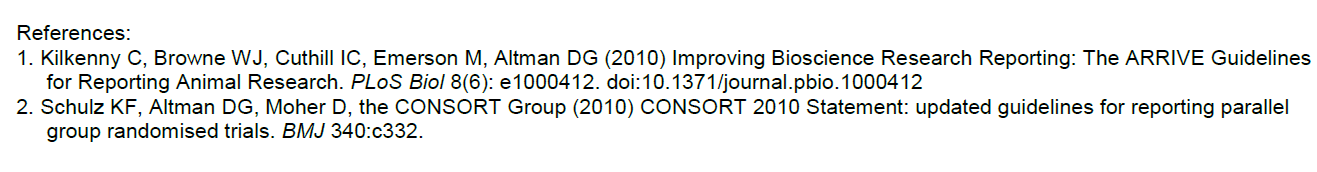


1. Abbott NJ, & Romero IA. (1996) Transporting therapeutics across the blood-brain barrier. Mol Med Today doi:10.1016/1357-4310(96)88720-X
2. Habgood MD, Begley DJ, Abbott NJ. (2000) Determinants of passive drug entry into the central nervous system. Cell Mol Neurobiol 20: 231–253.
3. Pardridge WM. (2005) The blood-brain barrier: bottleneck in brain drug development. NeuroRx 2: 3–14. doi:10.1602/neurorx.2.1.3
4. Pardridge WM. (2007) Drug targeting to the brain. Pharm Res 24: 1733–1744. doi:10.1186/1753-6561-2-s1-s37
5. Drago F, Caldwell JD, Pedersen CA, Continella G, Scapagnini U, et al. (1986) Dopamine neurotransmission in the nucleus accumbens may be involved in oxytocin-enhanced grooming behavior of the rat. Pharmacol Biochem Behav 24: 1185–1188. doi:10.1016/0091-3057(86)90168-1
6. Maurer TS, Debartolo DB, Tess DA, Scott DO. (2005) Relationship between exposure and nonspecific binding of thirty-three central nervous system drugs in mice. Drug Metab Dispos 33: 175–181. doi:10.1124/dmd.104.001222
7. Alam MI, Beg S, Samad A, Baboota S, Kohli K, et al. (2010) Strategy for effective brain drug delivery. Eur J Pharm Sci. doi:10.1016/j.ejps.2010.05.003
8. Choi JJ, Pernot M, Small SA, Konofagou EE. (2007) Noninvasive, transcranial and localized opening of the blood-brain barrier using focused ultrasound in mice. Ultrasound Med Biol 33: 95–104. doi:10.1016/j.ultrasmedbio.2006.07.018
9. Hynynen K, McDannold N, Vykhodtseva N, Jolesz FA. (2001) Noninvasive MR imaging-guided focal opening of the blood-brain barrier in rabbits. Radiology 220: 640–646. doi:10.1148/radiol.2202001804
10. Xie F, Boska MD, Lof J, Uberti MG, Tsutsui JM. (2008) Effects of Transcranial Ultrasound and Intravenous Microbubbles on Blood Brain Barrier Permeability in a Large NHP Model. Ultrasound Med Biol 34: 2028–2034. doi:10.1016/j.ultrasmedbio.2008.05.004
11. Tung Y-S, Marquet F, Teichert T, Ferrera V, Konofagou EE. (2011) Feasibility of noninvasive cavitation-guided blood-brain barrier opening using focused ultrasound and microbubbles in nonhuman primates. Appl Phys Lett 98: 163704. doi:10.1063/1.3580763
12. Olumolade O, Samiotaki G, Konofagou EE. (2012) Longitudinal Behavioral and Motor Control Studies of Repeated Focused Ultrasound Induced Blood-Brain Barrier Openings in Mice. 3^rd^ International symposium on Focused Ultrasound, Bethesda, MD, USA.
13. Sun T, Wang S, Acosta CJ, Konofagou EE. (2014) Cavitation characterization in the safety assessment of FUS-enhanced Blood-brain barrier opening. IEEE International Ultrasonics Symposium, Chicago, IL, USA.
14. Marquet F, Teichert T, Wu S-Y, Tung Y-S, Downs M, et al. (2014) Real-time, transcranial monitoring of safe blood-brain barrier opening in non-human primates. PloS One 9: e84310. doi:10.1371/journal.pone.0084310
15. McDannold N, Arvanitis CD, Vykhodtseva N, Livingstone MS. (2012) Temporary Disruption of the Blood-Brain Barrier by Use of Ultrasound and Microbubbles: Safety and Efficacy Evaluation in Rhesus Macaques. Cancer Res doi:10.1158/0008-5472.CAN-12-0128
16. Hom GJ, Bach TJ, Carroll D, Forrest MJ, Mariano MA, et al. (1999) Comparison of cardiovascular parameters and/or serum chemistry and hematology profiles in conscious and anesthetized rhesus monkeys (Macaca mulatta). Contemp Top Lab Anim Sci 38(2): 60-64.
